# Risk-driven security testing using risk analysis with threat modeling approach

**DOI:** 10.1186/2193-1801-3-754

**Published:** 2014-12-19

**Authors:** Maragathavalli Palanivel, Kanmani Selvadurai

**Affiliations:** Department of Information Technology, Pondicherry Engineering College, Puducherry, India

**Keywords:** Security testing, Risk analysis, System states, Risk-driven, Threat modeling, STRIDE, Test suite

## Abstract

Security testing is a process of determining risks present in the system states and protects them from vulnerabilities. But security testing does not provide due importance to threat modeling and risk analysis simultaneously that affects confidentiality and integrity of the system. Risk analysis includes identification, evaluation and assessment of risks. Threat modeling approach is identifying threats associated with the system. Risk-driven security testing uses risk analysis results in test case identification, selection and assessment to prioritize and optimize the testing process. Threat modeling approach, STRIDE is generally used to identify both technical and non-technical threats present in the system. Thus, a security testing mechanism based on risk analysis results using STRIDE approach has been proposed for identifying highly risk states. Risk metrics considered for testing includes risk impact, risk possibility and risk threshold. Risk threshold value is directly proportional to risk impact and risk possibility. Risk-driven security testing results in reduced test suite which in turn reduces test case selection time. Risk analysis optimizes the test case selection and execution process. For experimentation, the system models namely LMS, ATM, OBS, OSS and MTRS are considered. The performance of proposed system is analyzed using Test Suite Reduction Rate (TSRR) and FSM coverage. TSRR varies from 13.16 to 21.43% whereas FSM coverage is achieved up to 91.49%. The results show that the proposed method combining risk analysis with threat modeling identifies states with high risks to improve the testing efficiency.

**Electronic supplementary material:**

The online version of this article (doi:10.1186/2193-1801-3-754) contains supplementary material, which is available to authorized users.

## 1 Introduction

Testing is a process of identifying defects and checking the performance functionalities present in a system. The main aim of testing is to identify the results when a specific data is given as input. But the threats present in the system may lead to system malfunctioning. So security testing is done to identify the vulnerable states in the system. It is a type of software testing that intends to identify uncover vulnerabilities of the system and to determine whether its data and resources are protected from intruders or not. Security testing focuses on the related risks present in the system. It covers basic security concepts namely confidentiality, integrity, authentication, authorization, availability and non-repudiation.

The concepts of security are applicable to real-time systems and so models of the system are needed for better testing which indeed leads to Model-Based Security Testing. It relies on models of a System Under Test (SUT) and its environment. Model-Based Security Testing is a combination of four approaches namely security testing, risk-oriented testing, model-based testing and test automation. Risk-oriented testing uses risk analysis results in test case identification, selection and assessment to prioritize and optimize the testing process.

Test automation is the process of controlling the execution of test cases and comparing actual outcomes with predicted outcomes automatically. Security testing is mainly done to cover the basic security concepts and to make a system less vulnerable from attacks. It is important to identify the threats associated with the system which identify vulnerabilities in the system.

Threat modeling is a procedure to optimize security by identifying objectives and vulnerabilities and then defining counter measures to prevent or mitigate the effects of the threats present in the system. There are three approaches to threat modeling - they are attacker centric, software centric and asset centric. Attacker centric threat modeling starts with an attacker and evaluates their goals. Software centric threat modeling starts from the design of a system and attempts to step through a model of the system looking for various attacks against each element of the node. Asset centric threat modeling involves starting from assets entrusted to a system. Since threats associated with the system must be identified, software centric approach is suitable for MBST because the entire system design is to be processed for different types of attacks present in the system. There are different types of threat modeling processes which are used to identify threats and to identify stakeholder's risk. There are two different Microsoft threat modeling processes are STRIDE and DREAD. STRIDE is an acronym of six types of threats; Spoofing, Tampering, Repudiation, Information Disclosure, Denial of Service and Elevation of Privilege. It is used to identify both technical and non-technical threats. DREAD stands for Damage, Reproducibility, Exploitability, Affected users and Discoverability. It is used for rating threats and also for quantifying, comparing and prioritizing the amount of risks associated with each threat. There is another threat modeling framework, similar to STRIDE and DREAD, called TRIKE. It is mainly used to reduce stake holder's risk.

Risk analysis is the quantitative analysis of risk present in a system. Risk analysis is done based on the threat modeling results. Risk analysis is performed to find the vulnerable states that need to be tested. Risk Driven Security Testing (RST) and Test Driven Security Risk Analysis (TSR) are the two approaches of risk analysis. Security risk analysis is a specialized risk analysis approach in which information security risk associated with the potential threats will be evaluated. In RST, security testing is supported by security risk assessment in order to make security testing more effective. The aim is to focus the security testing process to carry out security tests on the most important parts of the System Under Test, and to execute only the selected test cases. In TSR security risk analysis is supported by security testing in order to develop and/or validate risk models. The objective of TSR is to strengthen the correctness of the security risk analysis models.

Risk analysis uses risk metrics namely risk probability, risk impact and risk threshold. Risk probability is the possibility that a risk can occur. Risk impact is the damage made by the risk when it occurs. Risk threshold is the maximum limiting value up to which the risk can be tolerated. The product of risk probability and risk impact identifies the vulnerability of risk associated with the state.

Test cases are selected based on the risk analysis results so that the states with the high probability of risk must be tested. Risk analysis optimizes the test case selection and execution process. Reduction in original test suite is represented using Test Suite Reduction Rate (TSRR). The reduced test suite is subjected to coverage criteria in order to identify its coverage percentage to the entire system model. Coverage is the measure of the degree to which the system is tested. There are a number of coverage criteria namely statement coverage, function coverage, branch coverage, condition coverage and many more. Transition coverage is taken as the performance metric since each system model is represented in extended finite state machine (EFSM).

Finally, security testing on risk analysis using STRIDE approach has been taken as a proposal to reduce the test suite size and to test the most vulnerable states in a system by using risk metrics. The system is also evaluated by parameters namely TSRR and transition coverage to enhance the performance. This paper is organized as follows: the section 2 discusses about the related work, section 3 describes about the proposed work followed by implementation, next section 4 analyses the results with the system description, section 5 concludes the paper followed by references.

## 2 Related work

Testing is the process of analyzing the result of a system for a particular test data. The test results can identify the errors present in the functionalities. But the risk associated with the system may also make a system defective and the type of testing which identifies and minimizes the defects is known as security testing. Risk is the possibility of attack to a system and the process of determining the vulnerabilities present is called Risk analysis.

Stallbaum et al. (2008) proposed a system known as RiteDAP, which generates test case based on activity diagrams and optimize those test cases based on risk (Olga and Vladik 2012). Roongruangsuwan and Daengdej 2005–2010; (Zimmermann et al. 2009) proposed a prioritization technique to optimize multiple test suites and test cases with same priority values. Priority based optimization is taken from this paper. Sabharwal et al. 2011; (Roongruangsuwan & Daengdej 2005–2010) proposed to optimize test case scenarios by identifying the critical path clusters using genetic algorithm. From this, path identification is learnt.

Papadakis and Malevris (Papadakis & Malevris 2013) proposed a framework for the automation of mutation testing method which uses dynamic search-based approaches for generating and evaluating mutation based test data. The concept of generating optimized test cases is learnt from this paper. Ogata and Matsuura (2013) introduced the new system model, Library Management System (LMS) for object-oriented analysis and design. This system model is included as one, in the data set. A new book evaluation methodology for utility management of university library has been discussed in (Yan et al. 2013); evaluation of LMS is learnt from this paper. In (Dessie 2014), dynamic modeling is used for medical applications.

In order to make a system less vulnerable to the risks, security testing and risk analysis is combined into two approaches namely: Risk-Driven Security Testing (RST) (Perueux 2013) and Test-Driven Security Risk Analysis (TSR). RST is a part of Risk-based testing which uses risk analysis results in test case identification and selection for optimizing the test process (Schieferdecker et al. 2011). It bridges the hierarchy between risk analysis and security testing (Grobmann et al. 2013) since the essential risk factors might be missed when risk analysis remains at high level (Sabharwal et al. 2011). TSR focuses mainly on risk analysis where testing process is carried out to validate risk models.

**Figure 1:**
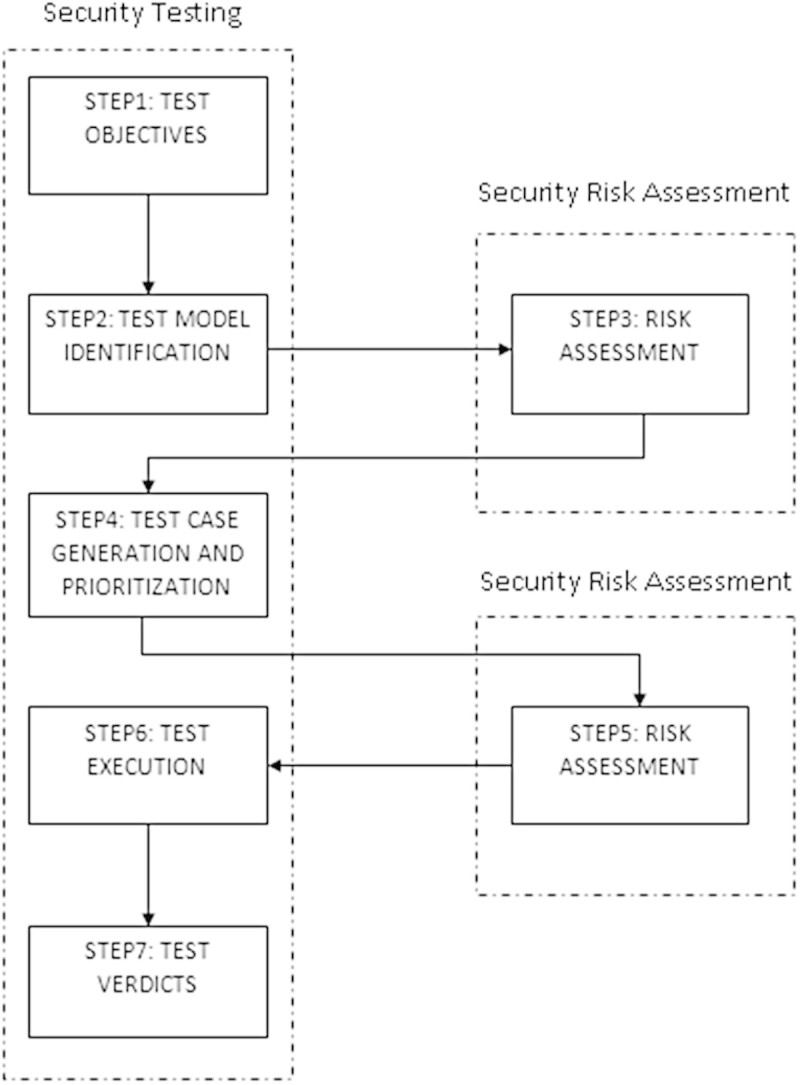
**Risk-Driven Security Testing (RST).**

Figure 1 shows the design of RST. RST is defined as Model-Based Security Testing (MBST) that uses risk analysis (Stijohann and Cuellar 2013) within the security testing process (Tim & Paul 2012). MBST is a special form of Model-based testing (MBT) (Erdogan & Stolen 2012) that focuses on the testing of security properties of a system (Omar et al. 2012). The first two steps deal with the identification of test objectives and model. It is followed by Risk assessment which identifies the risk associated with the system. The result from the risk assessment is used for test case generation and prioritization. It is again subjected to risk assessment and the significant test cases are executed.

**Figure 2:**
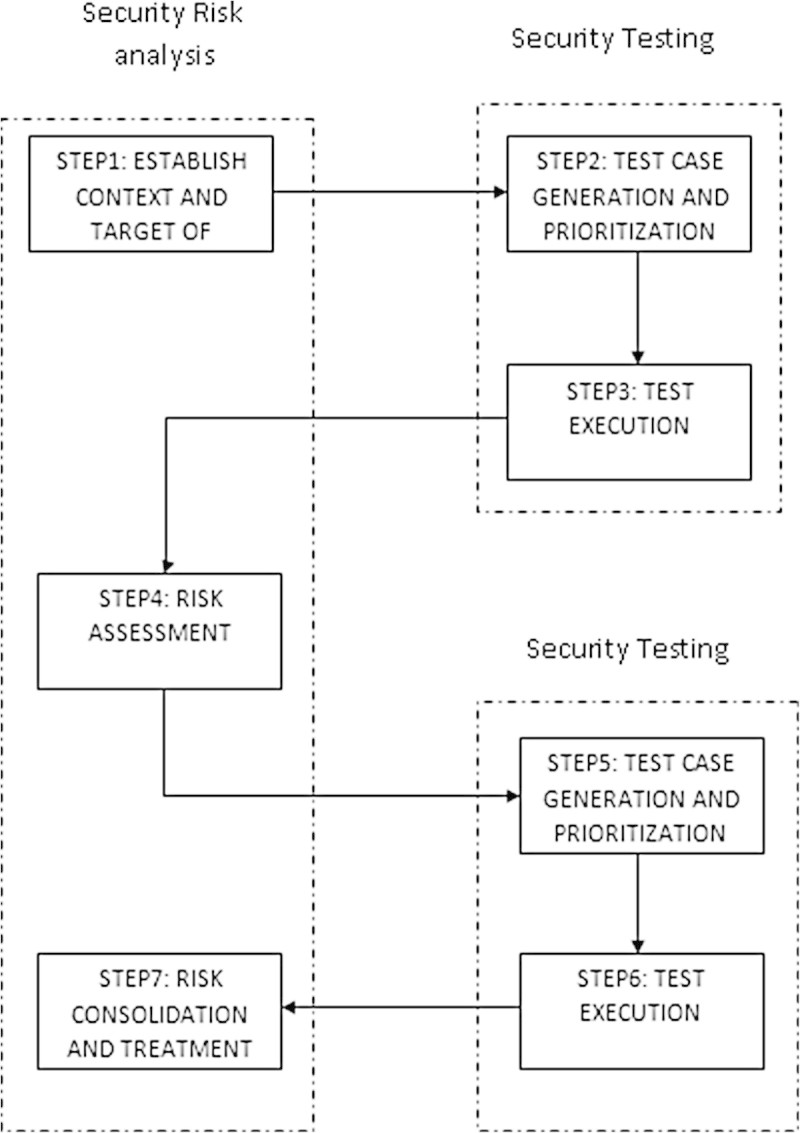
**Test-Driven Security Risk Analysis (TSR).**

Figure 2 shows the design of TSR. In TSR, risk analysis is supported by security testing in order to develop or validate risk models. The first step is to find the target system for risk analysis. It is followed by test case generation and execution for the development of risk models by identifying potential risks which is subjected to risk assessment. The testing process is again repeated to validate risk models which are then subjected to risk treatment.

In (Yan 2012) discussed risk-driven model-based security testing (RMST) and test-driven model-based security risk analysis (TMSR) which reduces risk factor by analyzing risk factor of models. RMST and TMSR are very much similar to RST and TSR. RMST and TMSR identifies whether testing is done to improve risk analysis or risk analysis is done to optimize testing process on each models.

Table 1 describes about the survey on risk analysis in security testing and its role in various Risk-Based Testing approaches.

**Table 1:** **Study on risk-based security testing**

Sl. no.	Title	Year	Techniques	Metrics	Systems/models used
1	Risk-driven Security Testing versus Test-driven Security Risk Analysis	Feb 15, 2012	Risk-driven security testing and Test-driven security risk analysis	Confidentiality, Integrity, Availability and Accountability.	Industrial Case Study
2	Baseline for Compositional Test-Based Security Risk Assessment	Jan 31, 2013	Table based risk assessment technique	Risk identification, Risk Analysis, Risk Evaluation and Risk Treatment	Common Vulnerability Scoring system
3	Baseline for Compositional Risk-Based	Jan 31, 2013	Risk-based vulnerability testing	Severity, Testability, Uncertainty, reusability	Scalable network system
	Security Testing				
4	Risk-based Statistical Testing: A Refinement based	May 2009	Model-based statistical testing, Markov chain test models	Safety Integrity Level (SIL)	Critical systems like fire alarm, railway control system
	Approach to the Reliability Analysis of Safety-Critical Systems				
5	Effort-dependent technologies for multi-domain risk-based security testing	Sept 27, 2010	Light weight risk and security testing	Proof-of-Performance, Proof-of-Concept, Proof-of-Existence	Security Audit of Supplier services, Maintaining security in virtual organization

Mitrabinda and Durga Prasad 2013; (Grobmann & Viehmann 2013) proposed a state-based risk assessment methodology (Diamonds Project 2010–2014) at the analysis and design stage of Software DevelopmentLife Cycle. The parameters used for risk assessment are complexity and severity. The risk for a scenario is estimated based on the risk of interacting components in various states within the scenario and StateCOllaboration TEst Model (SCOTEM) of the scenario.

## 3 Proposed system

A methodology for testing the systems using risk based approach named STRIDE has been introduced in analysis of security threats. By combining the risk analysis with threat modeling approach, risk based testing is performed.

### 3.1 Proposed work

**Figure 3:**
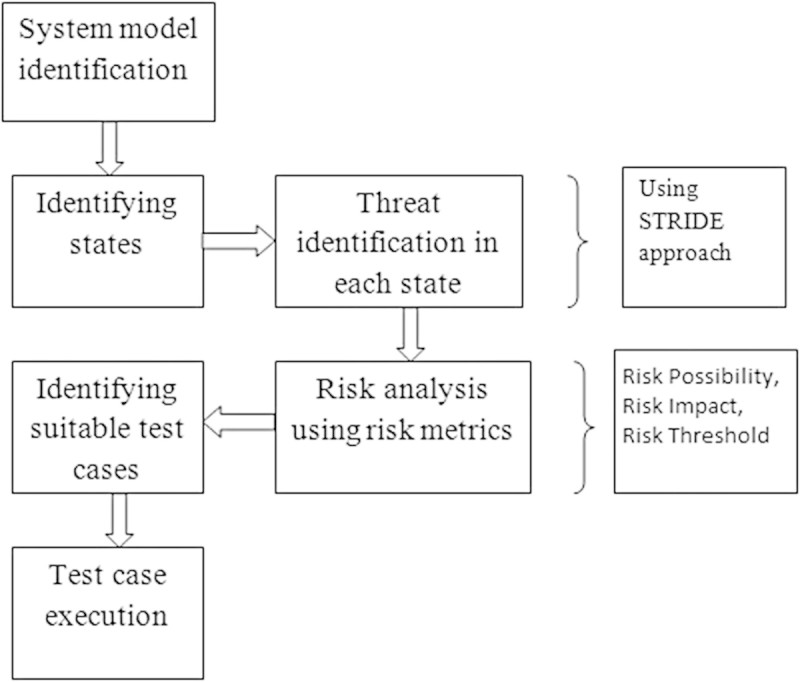
**Overall system design.**

The proposed Security Testing system mainly based on two concepts namely Risk Analysis and Threat Modeling approach. Threat modeling is based on the notion that any system or organization has assets of value worth protecting, these assets have certain vulnerabilities, internal or external threats exploit these vulnerabilities in order to cause damage to the assets, and appropriate security countermeasures exist that mitigate the threats. The different states and transitions present in the system model are defined by EFSM diagram and what are all possible threats for each state is found using STRIDE approach. STRIDE threat model is used to identify technical and non- technical threats associated with the states of the system. STRIDE stands for:

- Spoofing identity: An example of identity spoofing is illegally accessing and then using another user's authentication information, such as username and password.
- Tampering with data: Data tampering involves the malicious modification of data. Examples include unauthorized changes made to persistent data, such as that held in a database, and the alteration of data as it flows between two computers over an open network, such as the Internet.
- Repudiation: Repudiation threats are associated with users who deny performing an action without other parties having any way to prove otherwise—for example, a user performs an illegal operation in a system that lacks the ability to trace the prohibited operations. Non-repudiation refers to the ability of a system to counter repudiation threats. For example, a user who purchases an item might have to sign for the item upon receipt. The vendor can then use the signed receipt as evidence that the user did receive the package.
- Information disclosure: Information disclosure threats involve the exposure of information to individuals who are not supposed to have access to it—for example, the ability of users to read a file that they were not granted access to, or the ability of an intruder to read data in transit between two computers.
- Denial of service: Denial of service (DoS) attacks deny service to valid users—for example, by making a Web server temporarily unavailable or unusable. You must protect against certain types of DoS threats simply to improve system availability and reliability.
- Elevation of privilege: In this type of threat, an unprivileged user gains privileged access and thereby has sufficient access to compromise or destroy the entire system. Elevation of privilege threats include those situations in which an attacker has effectively penetrated all system defenses and become part of the trusted system itself, a dangerous situation indeed.
  Risk Analysis is carried out for each state based on threats and their risk values. The risks associated with various threats are identified using risk parameters like Risk possibility, Risk threshold and Risk impact. Based on Risk Analysis results, the more vulnerable test cases are identified. The overall system design is shown in Figure 3.

The modules in the system are as follows:

1. State representation module.
2. Threat modeling module.
3. Risk analysis module.
4. Test case selection module.

### 3.2 State representation module

**Figure 4:**
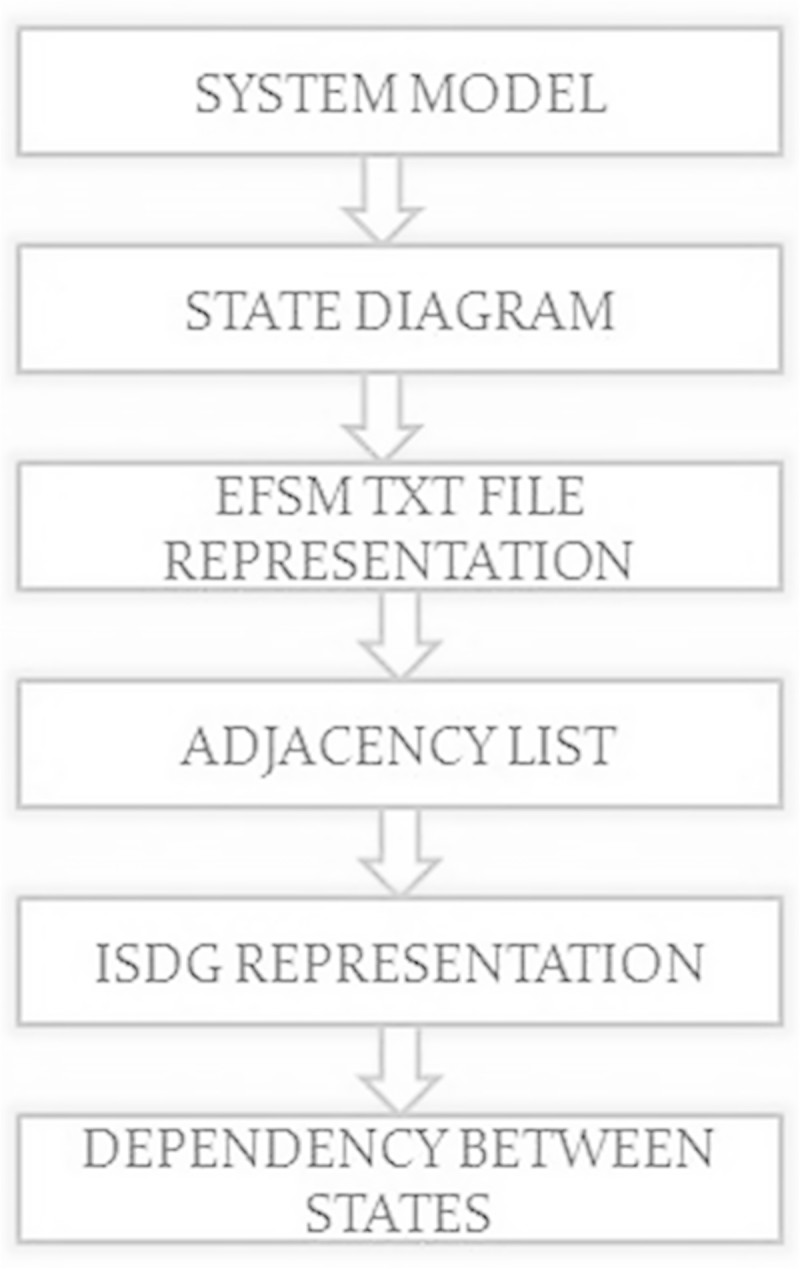
**Workflow diagram for state representation module.**

The system model is chosen and the states are defined and it is depicted using EFSM diagram then input is represented using EFSM txt file which contains all possible transitions and the workflow is shown in Figure 4. Transition refers to path between the start state and end state. After that the adjacency list is obtained from the model and the dependency between the states is found using adjacency matrix. If dependency between states is high, then the system is highly vulnerable.

### 3.3 Threat modeling module

**Figure 5:**
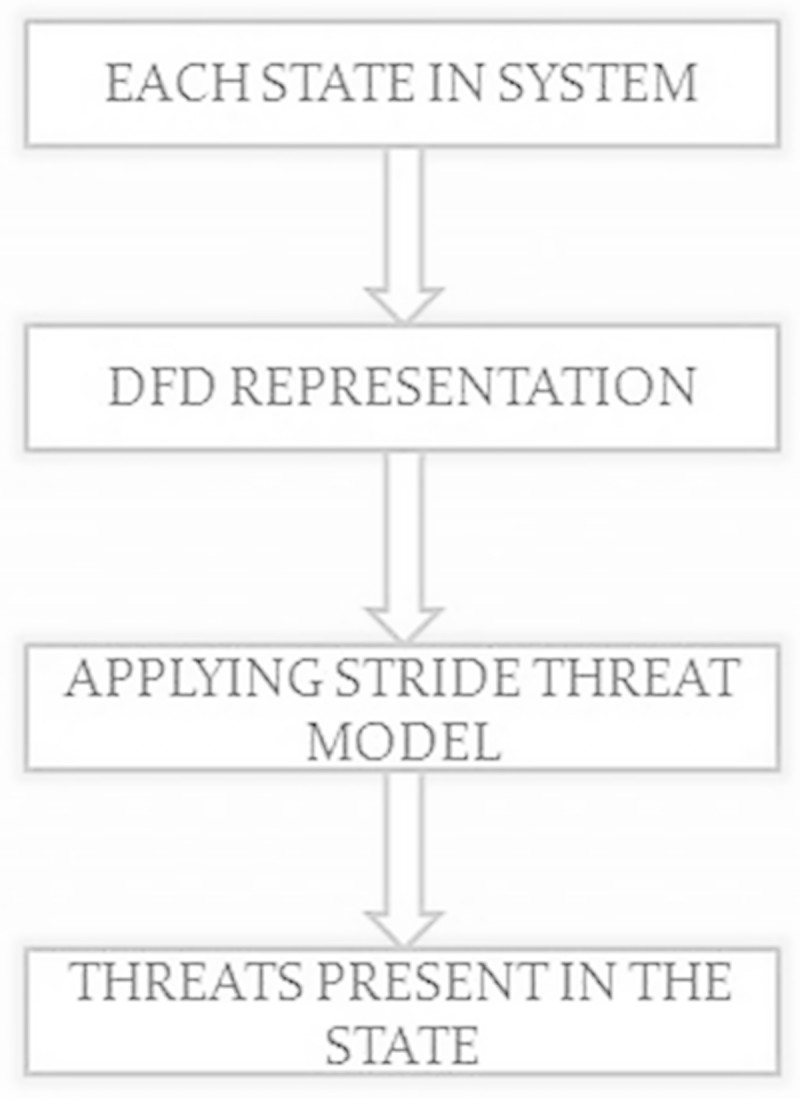
**Workflow diagram for threat modelling.**

In the system model where all states and its corresponding transitions are known the data flow diagram based on the states and process is drawn which depicts the flow of data in current system shown in Figure 5. It is used to identify the threats present in the system. STRIDE thread model identify six attacks namely Spoofing, Tampering, Repudiation, Information Disclosure, Denial of Service, and Elevation of Privilege. This model checks out of these attacks which one will be more prone to the system.

#### 3.3.1 Data flow diagrams

Data flow diagrams (DFDs) are typically used to graphically represent a system model. DFD consists of four elements namely data flow, data store, process and interactor.

Table 2 describes about the threats covered by the each element in the data flow diagram representation of the system model.

**Table 2:** **Applying STRIDE threat modeling**

Element	Spoofing	Tampering	Repudiation	Information disclosure	Denial of service	Elevation of privilege
Data Flow		X		X	X	
Data Store		X		X	X	
Process	X	X	X	X	X	X
Interactor	X		X			

X- Threats Covered.

### 3.4 Risk analysis module

**Figure 6:**
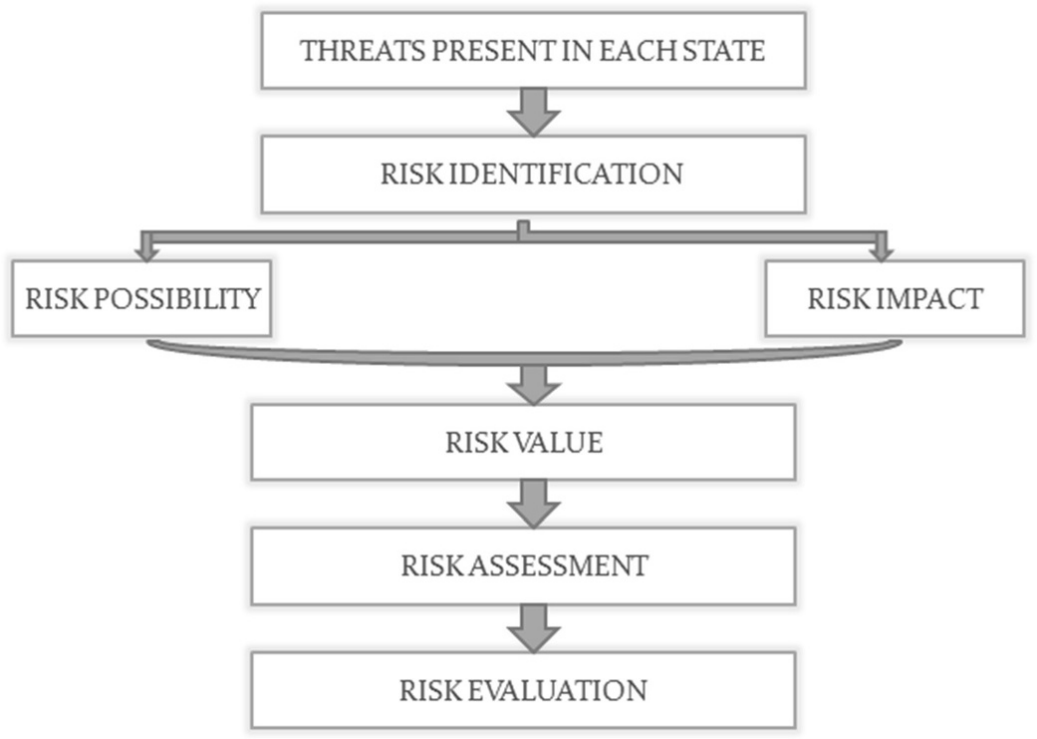
**Workflow diagram for risk analysis.**

In this module, the states which have threats are taken and it is sent for risk analysis. It is a process step which analyzes and identifies risk based on parameters known as Risk possibility, Risk Impact and Risk Threshold and is shown in Figure 6. Risk Possibility refers to how much the system is vulnerable. It tells whether system will be attacked by a threat or not. Risk Impact refers to effect of risk on other states in system model. It depends on dependency matrix which tells how much a certain risk will affect the system. Risk Value refers to product of Risk Possibility and its impact. Based on the value, risk will be compared with risk threshold and such state will be assigned with high, medium and low risks.

#### 3.4.1 Risk calculation

Riskateachstate=RiskPossibility*RiskImpact

where:

- Risk Possibility = Possibility of attacks in each state
- Risk Impact = Number of states affected with impact of risk in each state
- Risk possibility depends on Data Flow Diagram representation of risks which in turn helps to identify risks present in the system.
- Risk Impact depends on the dependency between the states in the system.
- Risk Threshold is the range of risk value upto which risk is tolerable. Risk threshold value varies for various systems.

### 3.5 Test case selection module

**Figure 7:**
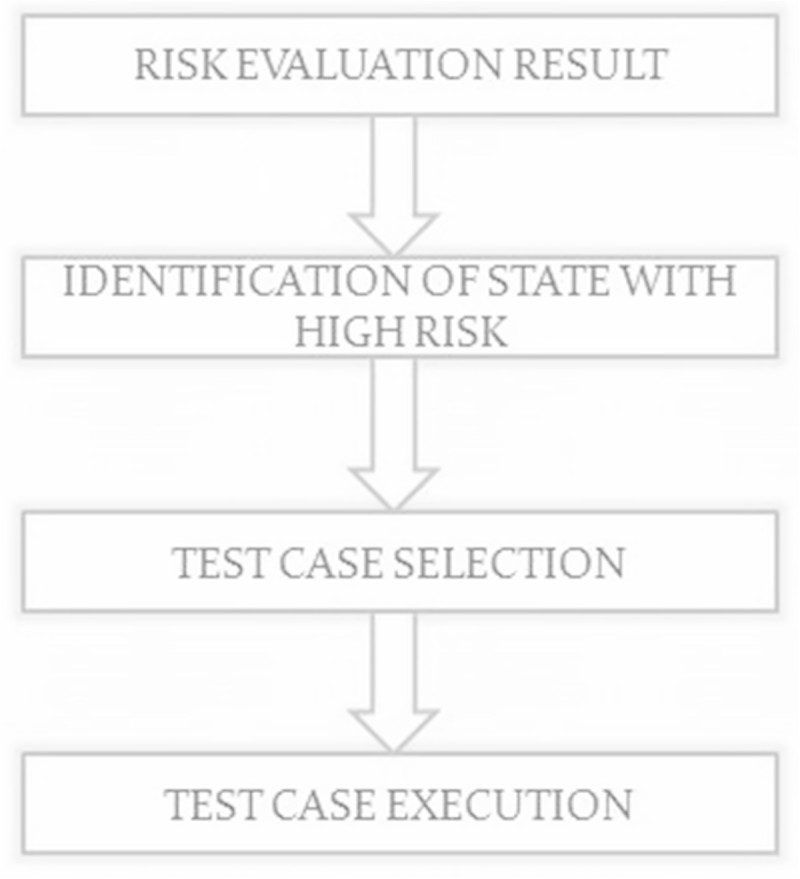
**Workflow diagram for test case selection and execution.**

The module is used to select which test cases have high vulnerability and test them under various attacks. Based on risk evaluation results the states will be grouped apart as high, medium and low based on their risk values. Then the possible test cases which contain many transitions involving states involving states with high risk are selected and the test cases are executed. In each state, test cases selection and execution flow is shown in Figure 7.

## 4 Results and discussion

The system models considered for experimentation are Library Management System (LMS), Automated Teller Machine (ATM), Online Banking System (OBS), Online Shopping System (OSS) and Movie Ticket Reservation System (MTRS) to evaluate the performance of risk analysis and threat modeling. These models are mentioned under the data set description.

### 4.1 Data set description

The following are EFSM models considered for the experimentation.

**Figure 8:**
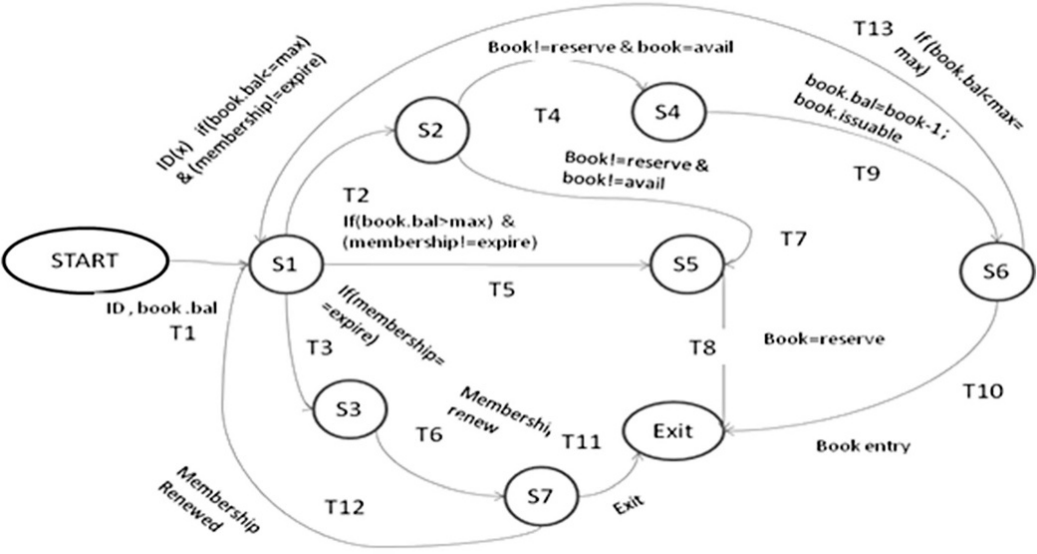
**EFSM for Library Management System (LMS).**

**Figure 9:**
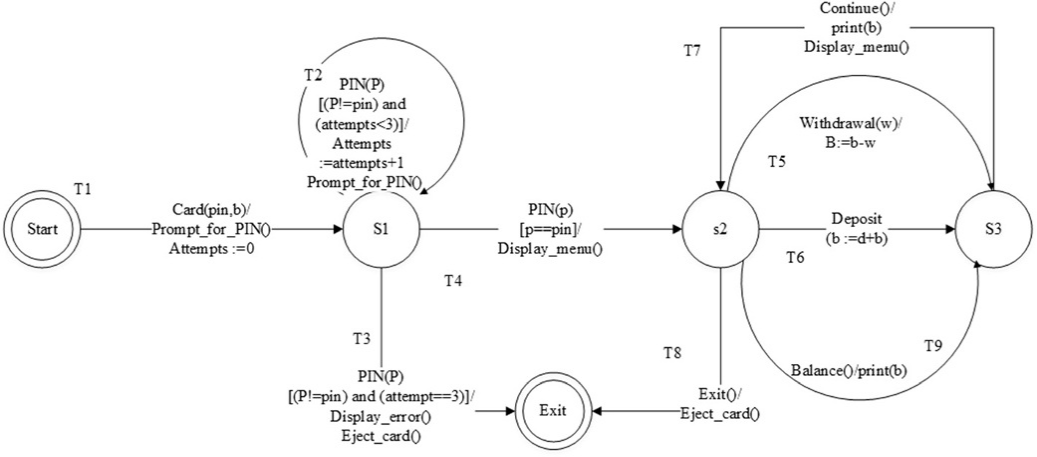
**EFSM for Automated Teller machine (ATM).**

**Figure 10:**
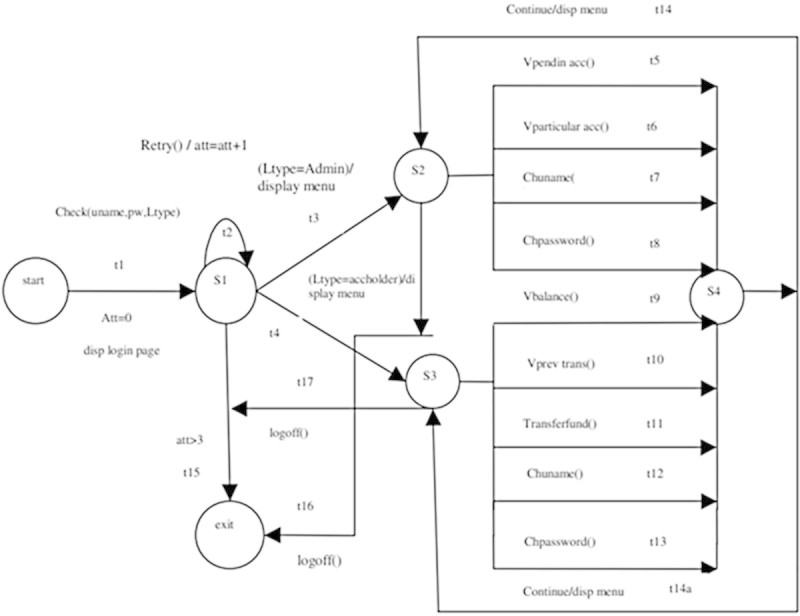
**EFSM for Online Banking System (OBS).**

**Figure 11:**
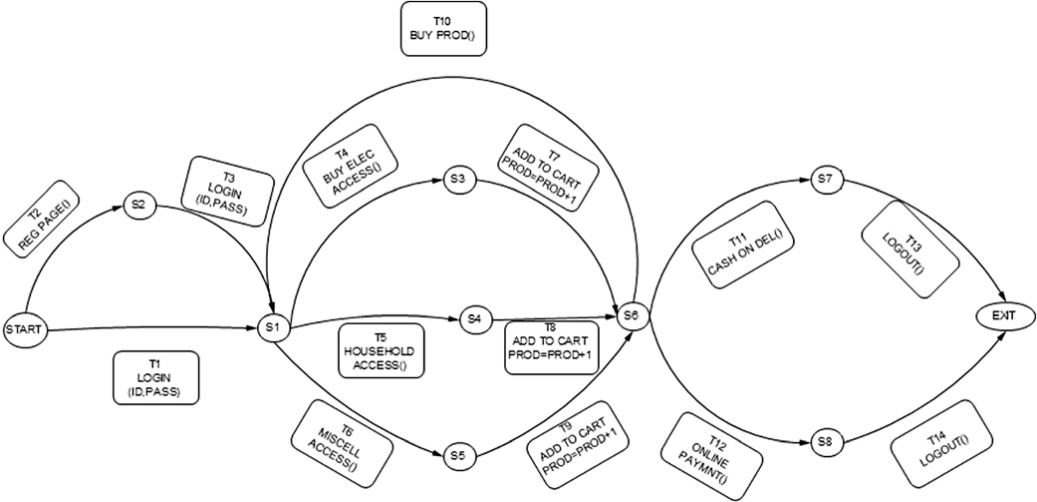
**EFSM for Online Shopping System (OSS).**

**Figure 12:**
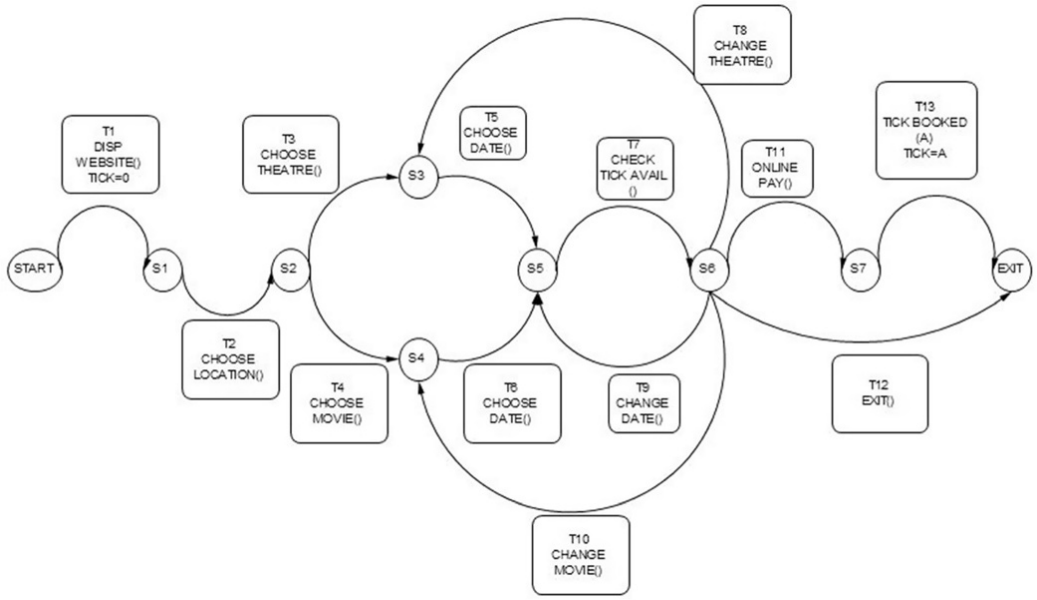
**EFSM for Movie Ticket Reservation System (MTRS).**

- **LMS**
  The EFSM representation of LMS consists of 7 states and 13 transitions which are shown in Figure 8. The main operations involved in LMS are renewal of membership, issue of books and reservation of books. LMS starts with checking of ID and the number of books borrowed by each user. If ID is already expired then the user has to renew membership to borrow or reserve books. Each user can borrow a maximum 4 books. If the user exceeds the maximum limit or a book needed by the user is already issued then reservation of the book is only allowed. A user may borrow a book only if the book is available for issue and the user has not reached the maximum limit.
- **ATM**
  The EFSM representation of ATM system consists of 3 states and 9 transitions shown in Figure 9. The main operations in ATM system are checking of balance, withdrawal and deposit of money. ATM system initiates with prompting of pin number from user. If a user enters incorrect pin number for more than 3 times, the card will be automatically ejected. If a user is authorized, then the user may withdraw or deposit money to a maximum of two times during the same transaction.
- **OBS**
  The EFSM representation of OBS consists of 4 states and 18 transitions shown in Figure 10. OBS can be accessed by admin or the account holder. Admin can view the account of any user or any pending account, change username and password for any user and also be able to view the balance amount in an account. Account holder can view previous transaction, transfer funds to another account and change username and password. A user has to enter the login credentials correctly within 2 attempts.
- **OSS**
  The EFSM representation of OSS consists of 8 states and 14 transitions shown in Figure 11. OSS allows the user to buy electronics accessories, household accessories and other miscellaneous products over internet. A user needs to create an account to purchase products. The user may use the same account for future purchases. For every login, the user may purchase to a maximum of 3 products. The payment might be online payment or cash on delivery.
- **MTRS**
  The EFSM representation of MTRS consists of 7 states and 13 transitions shown in Figure 12. MTRS allows a user to reserve movie tickets based on location, theatre, movie and date. The user has to pay money online for reservation of tickets and has to provide personal details for authentication. The required number of tickets can be reserved by searching over various theatres for various films.

### 4.2 Description of risk value calculation and risk metrics

In order to find the number of risks present in each state of a system the risk value is calculated using the risk metrics.

#### 4.2.1 Risk value calculation

Riskpresentineachstate=RiskPossibility*RiskImpact

#### 4.2.2 Risk metrics

1. Risk Possibility is the possible threats present in each state according to STRIDE.
2. Risk Impact is the number of states affected due to the impact of a threat on a state.
3. Risk Threshold is the range of risk value up to which risk is tolerable. Risk threshold helps to identify the states with low, medium and high risks.

### 4.3 Description of performance parameters

1. The test suite reduction rate (TSRR) is defined as the ratio of the number of test cases removed from the original test suite to the number of test cases in the original test suite.
  $$\mathrm{TSRR}=\frac{\left|T|‒|\mathrm{Tred}\right|}{\left|T\right|}*100\%$$
  where:
  |T| = Number of test cases in the original suite and
  |Tred| = Number of test cases in the minimized/reduced suite
2. The FSM coverage is defined as the ratio of the number of transitions in reduced test suite to the number of transitions in original test suite.
  $$\begin{array}{ll} FSM\;\mathrm{Coverage}\;\left(\%\right)= & \frac{\mathrm{No}.\;\mathrm{of}\;\mathrm{transitions}\;\mathrm{in}\;\mathrm{reduced}\;\mathrm{test}\;\mathrm{suite}}{\mathrm{No}.\;\mathrm{of}\;\mathrm{transitions}\;\mathrm{in}\;\mathrm{original}\;\mathrm{test}\;\mathrm{suite}}\\ {}*100 \end{array}$$

### 4.4 Result analysis

**Figure 13:**
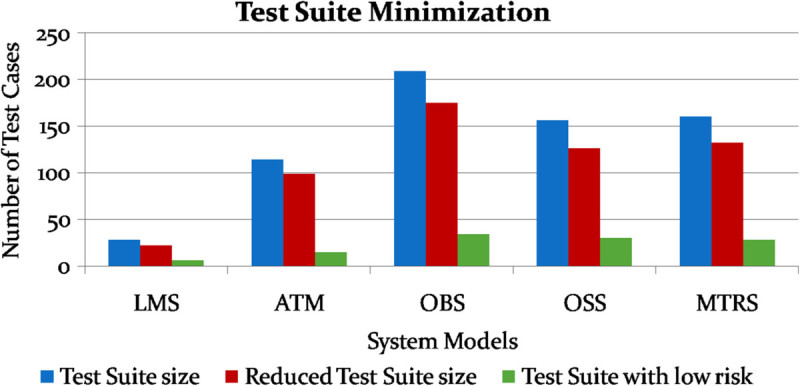
**Comparison of various test suite sizes for each system model.**

Test suite minimization depends on risk analysis results. Test cases with low risk are removed from the original test suite. Figure 13 shows the comparison of various test suite sizes for each system model. Test suite reduction rate (TSRR) refers to the number of test cases with low risk in the overall test suite. It is observed that the TSRR value varies from 16.27 to 21.43 for the system models considered which shows that the proposed system identifies more risks present in a system. From Table 3, it is also observed that the system with less number of states has high reduction rate since dependency between the states will be high.

**Table 3:** **Results of performance parameters**

S. no.	System models	No. of states	No. of transitions	Original test suite	Reduced test suite	Test Suite Reduction Rate (TSRR) in %	No. of transitions covered		FSM coverage in %
							Before risk analysis	After risk analysis	
1	LMS	7	13	28	22	21.43	224	197	87.95
2	ATM	3	9	114	99	13.16	1011	925	91.49
3	OBS	6	18	209	175	16.27	1665	1468	88.17
4	OSS	8	14	156	126	19.23	1614	1386	85.87
5	MTRS	7	13	160	132	17.5	2152	1882	87.45

**Figure 14:**
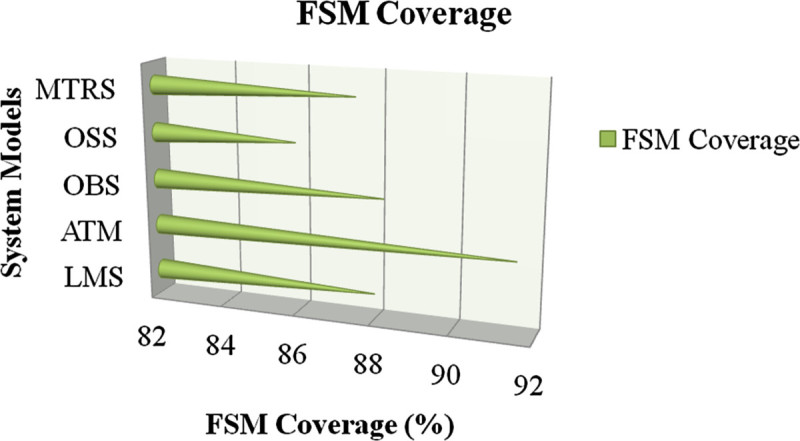
**Comparison of FSM coverage for system models.**

FSM coverage is a measure of degree to which the system model represented in EFSM is tested by a particular test suite. FSM coverage should be more for better testing results. FSM coverage value is calculated by the ratio of number of transition covered in original test suite to the number of transitions covered in reduced test suite. From the results, it is observed that the coverage value varies from 85.87 to 91.49 for the system models considered which justifies that the reduced test suite of each system model contains effective test cases. Figure 14 shows the comparison of the FSM coverage value for different system models. The quantitative results of performance parameters are tabulated and represented in Table 3.

**Figure 15:**
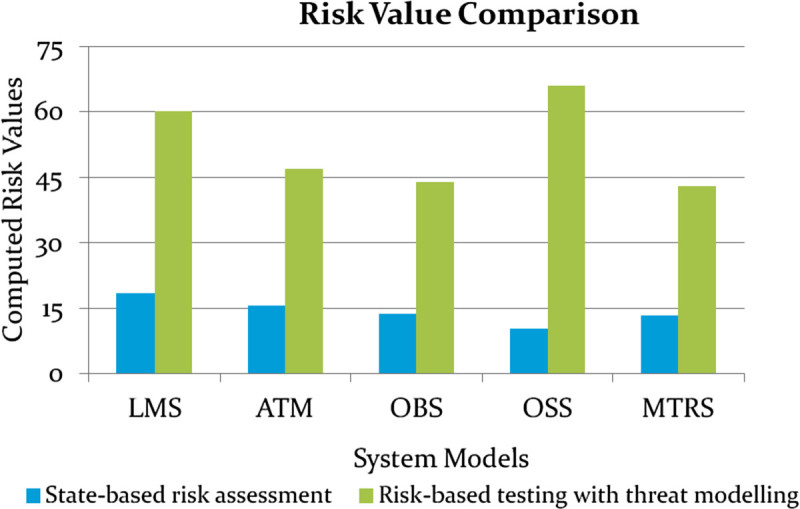
**Comparison of risk values.**

The quantitative analysis of risk performed by the proposed system is compared with the existing system. Existing system does not include threat modeling in risk analysis and uses complexity and severity as risk parameters. On the contrary, proposed system applies STRIDE threat modeling in risk analysis and uses risk possibility, risk impact and risk threshold as risk parameters. It is observed that the quantitative risk value calculated using proposed system is approximately three times better than the existing system. Figure 15 shows the comparison of quantitative risk values between existing and proposed system for each system model.

## 5 Conclusion

Thus, the proposed system applying STRIDE threat modeling in risk analysis for identifying risks present in a system and reducing test cases based on risk analysis results performed better than the existing system. Existing system uses a state-based risk assessment methodology which does not include threat modeling. It is observed that the proposed system produced three times better results in identifying risks present in a system compared to the existing system using risk metrics namely risk possibility, risk impact and risk threshold. Risk analysis helps in the identification and elimination of test cases with comparatively low risks from the test suite. The proposed system is analyzed using performance parameters namely TSRR and FSM coverage. TSRR varied from 13.16to 21.43 whereas a maximum of 91.49% FSM coverage is achieved. The results from the performance parameters also justifies that the proposed system effectively identifies risks and the reduced test suite provides better testing of the system models.

## Authors’ original submitted files for images

Below are the links to the authors’ original submitted files for images. Authors’ original file for figure 1 Authors’ original file for figure 2 Authors’ original file for figure 3 Authors’ original file for figure 4 Authors’ original file for figure 5 Authors’ original file for figure 6 Authors’ original file for figure 7 Authors’ original file for figure 8 Authors’ original file for figure 9 Authors’ original file for figure 10 Authors’ original file for figure 11 Authors’ original file for figure 12 Authors’ original file for figure 13 Authors’ original file for figure 14 Authors’ original file for figure 15
