# Supplementary figures and images for: Risk-driven security testing using risk analysis with threat modeling approach
Source: Springerplus. 2014 Dec 19;3:754. doi: 10.1186/2193-1801-3-754 (PMC4320241; doi:10.1186/2193-1801-3-754)

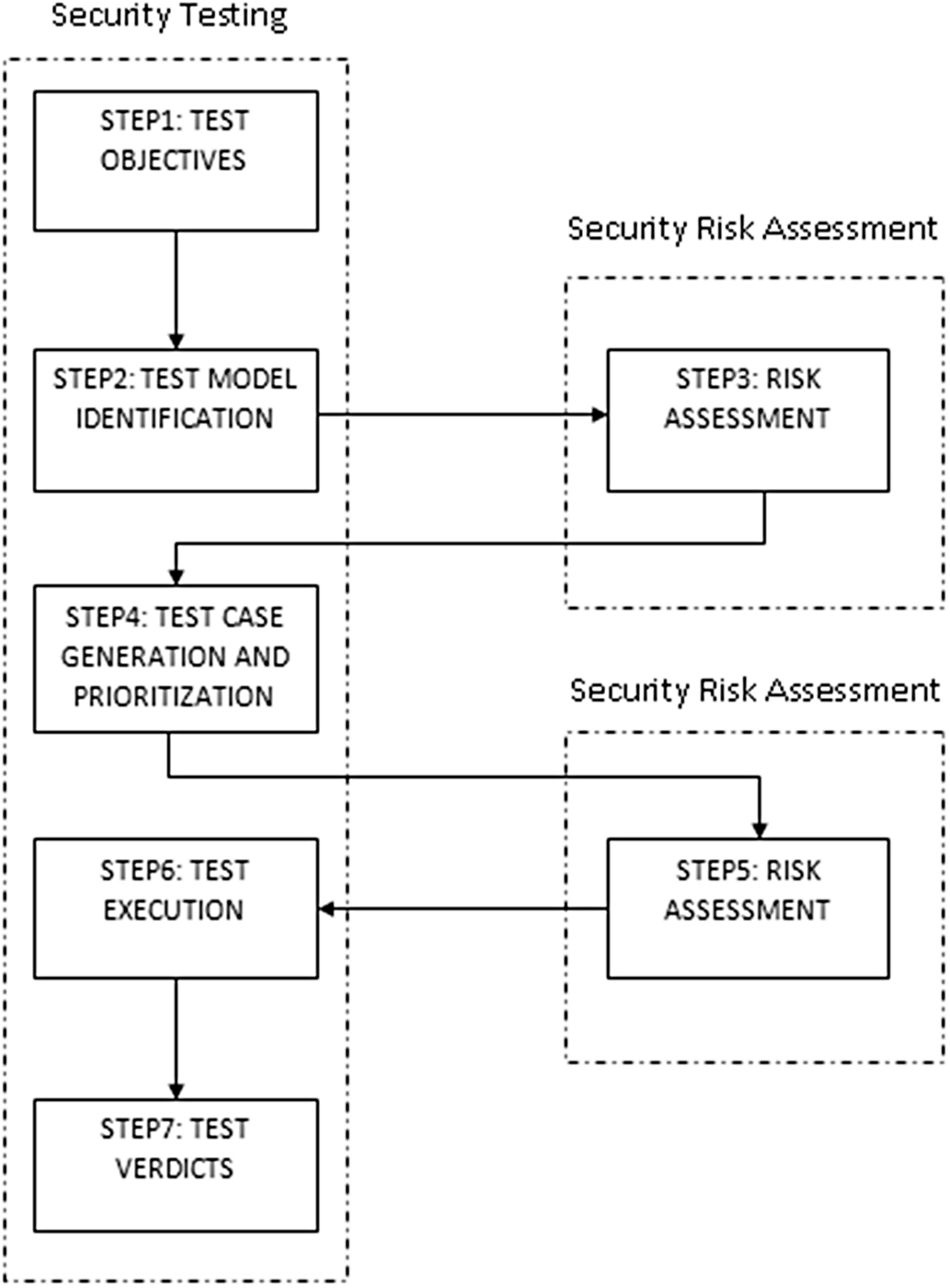

Supplement: Supplementary file 1 — Authors’ original file for figure 1 [file 40064_2014_1515_MOESM1_ESM.tiff]

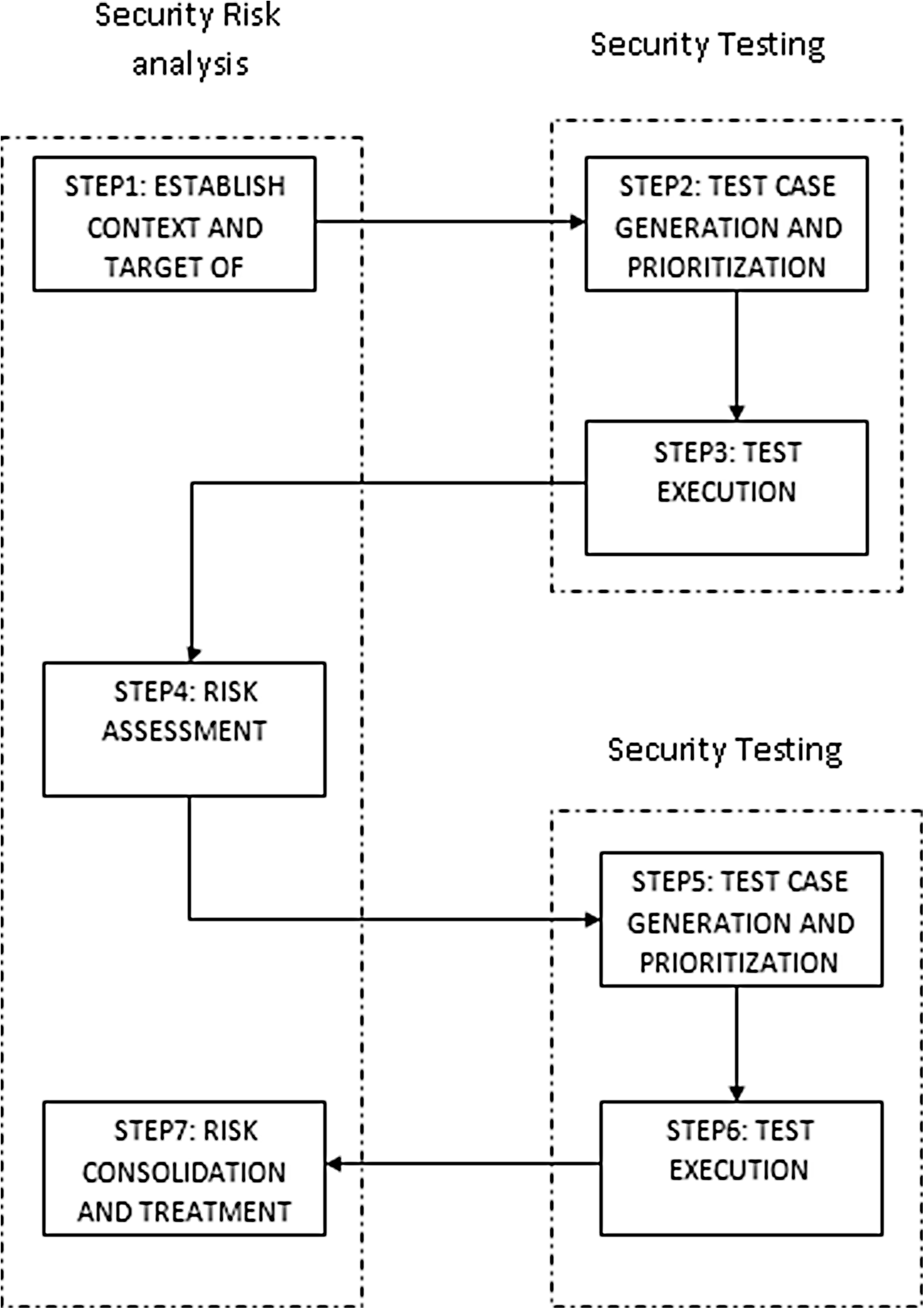

Supplement: Supplementary file 2 — Authors’ original file for figure 2 [file 40064_2014_1515_MOESM2_ESM.tiff]

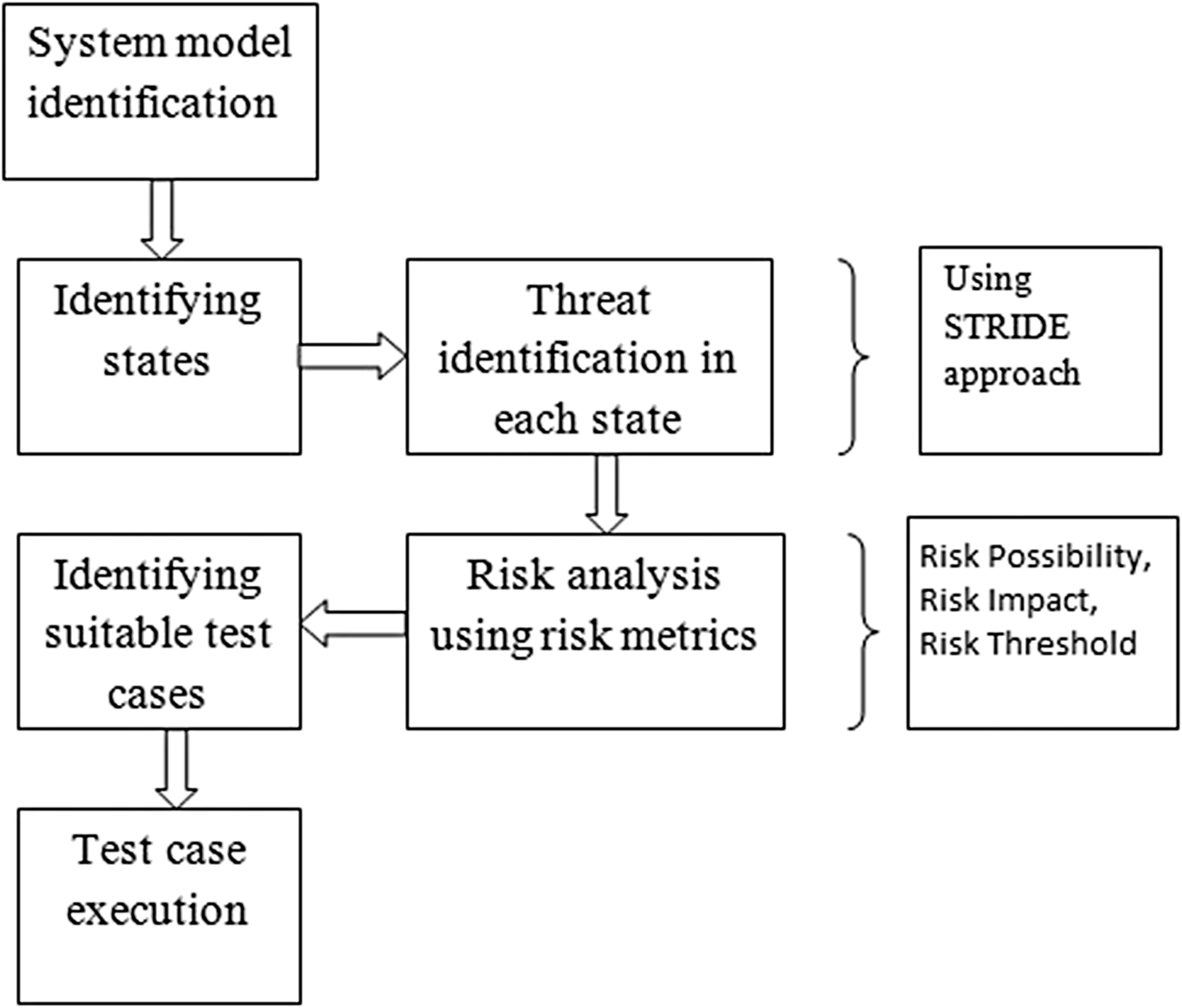

Supplement: Supplementary file 3 — Authors’ original file for figure 3 [file 40064_2014_1515_MOESM3_ESM.tiff]

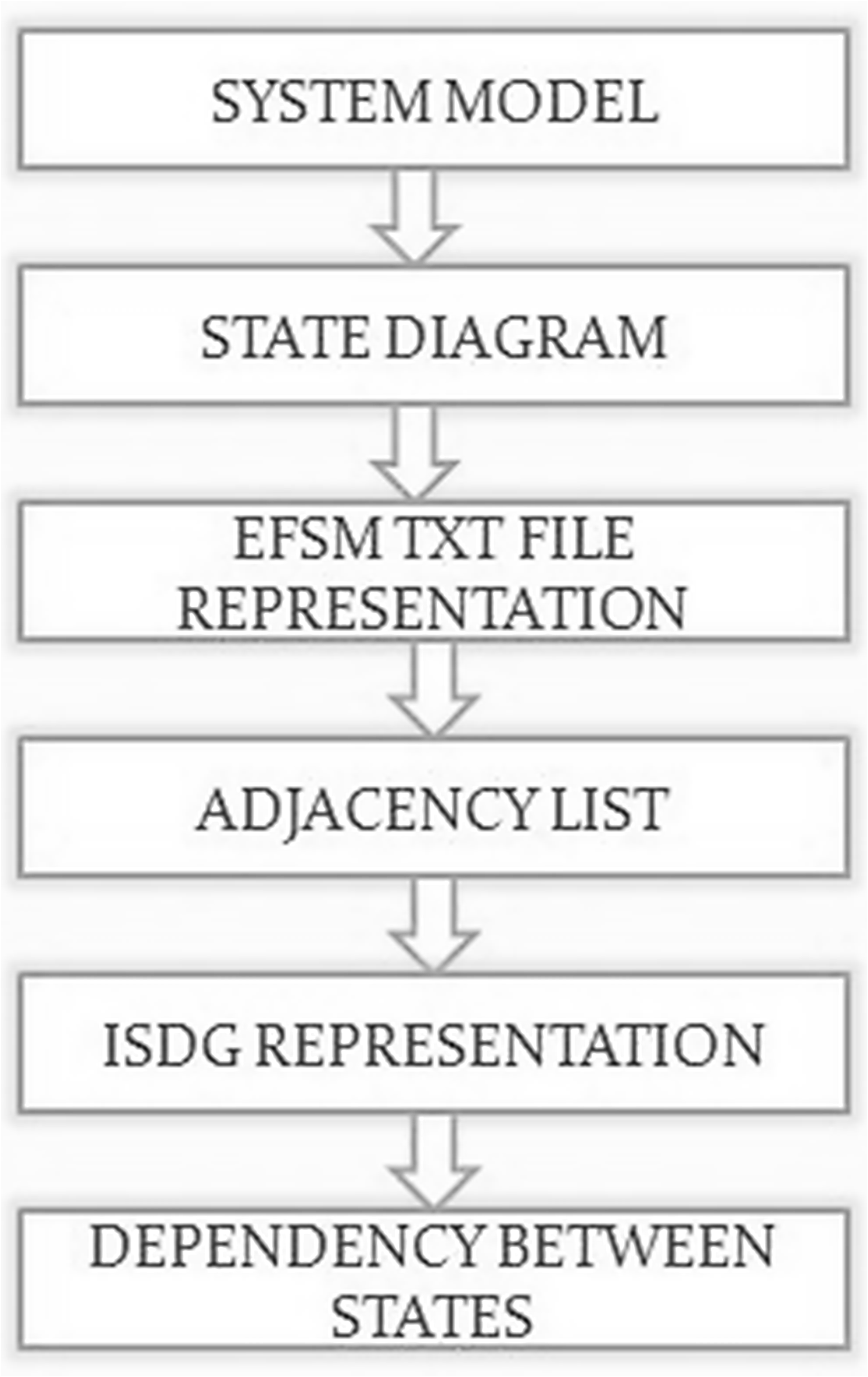

Supplement: Supplementary file 4 — Authors’ original file for figure 4 [file 40064_2014_1515_MOESM4_ESM.tiff]

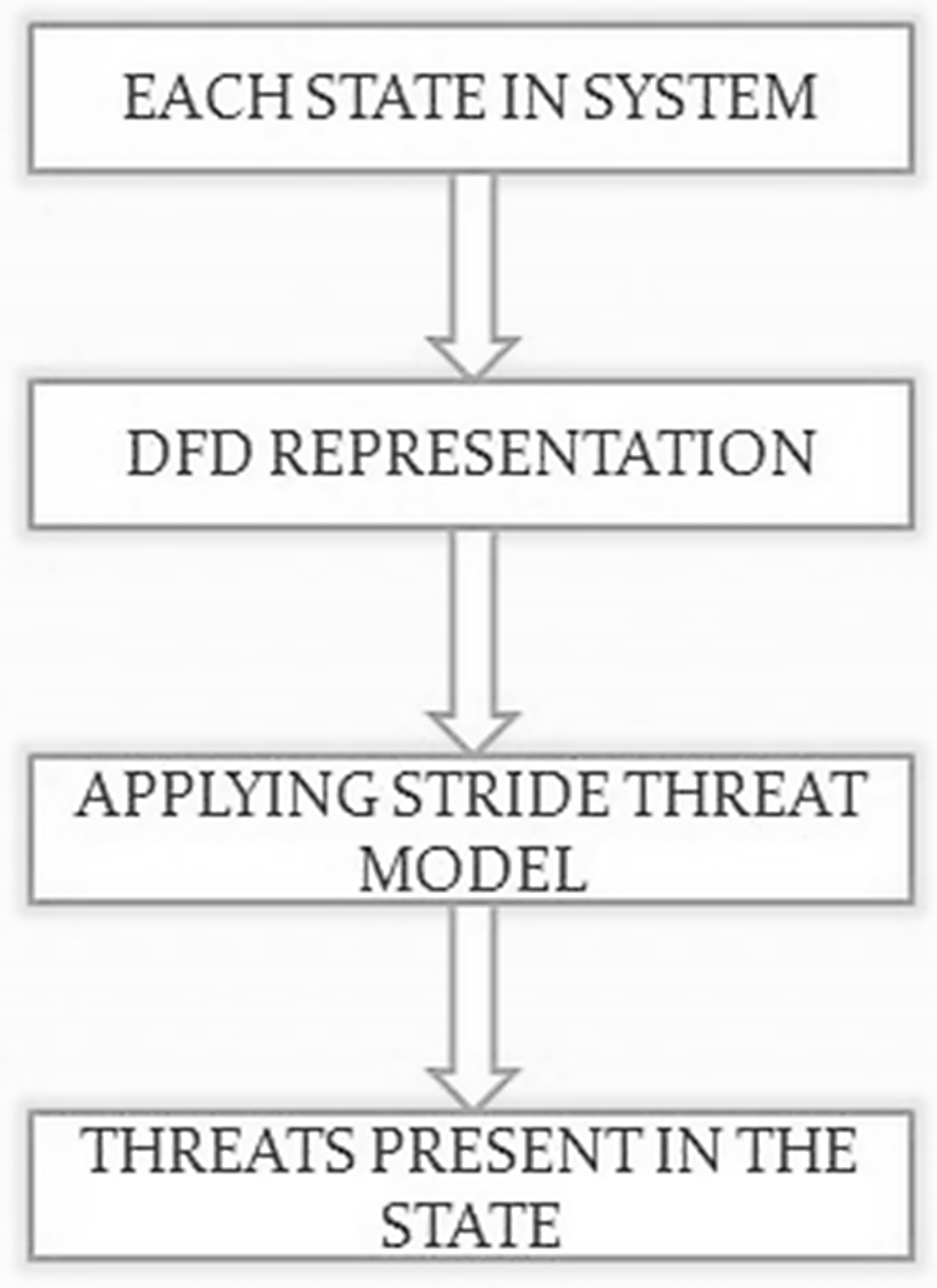

Supplement: Supplementary file 5 — Authors’ original file for figure 5 [file 40064_2014_1515_MOESM5_ESM.tiff]

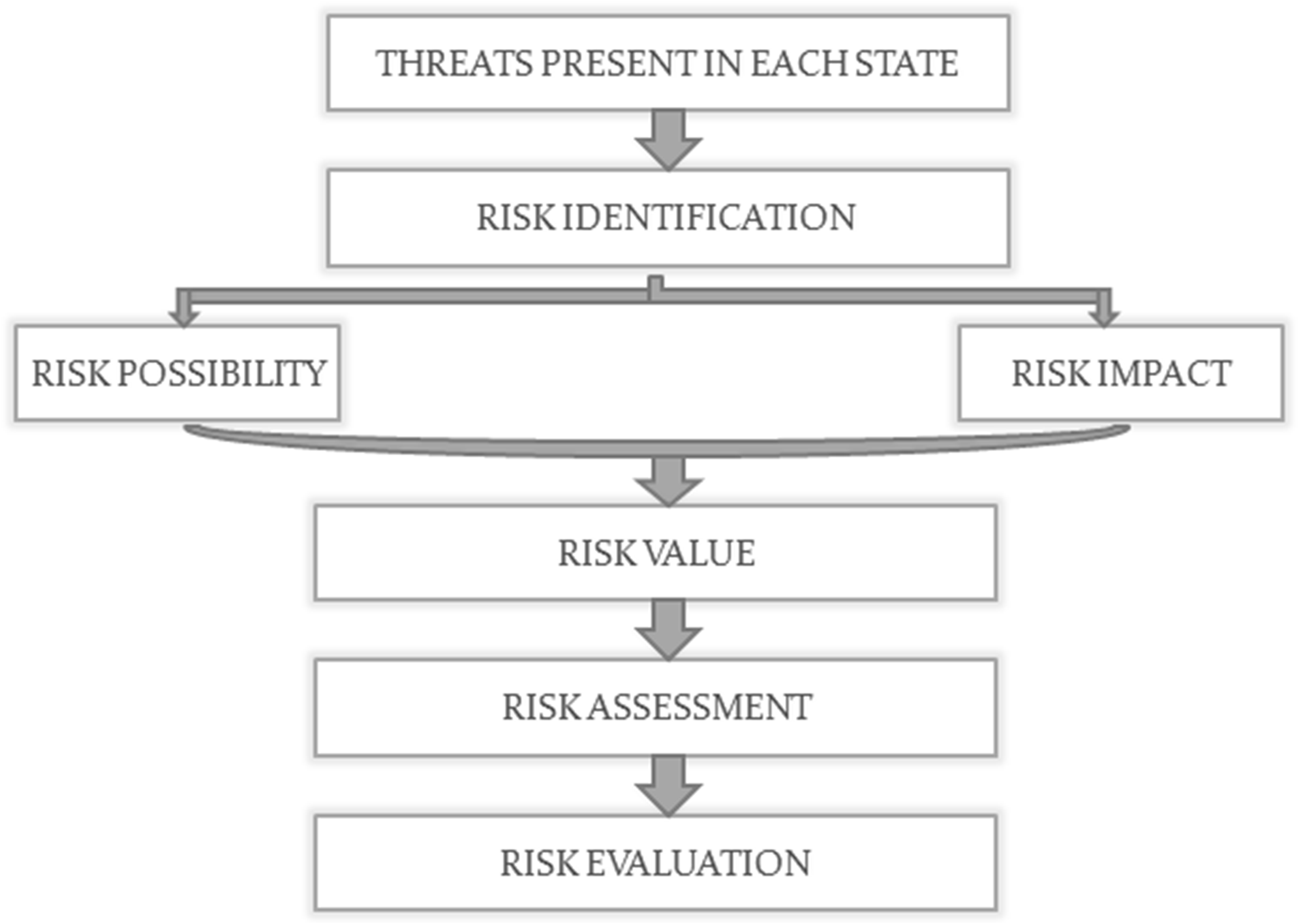

Supplement: Supplementary file 6 — Authors’ original file for figure 6 [file 40064_2014_1515_MOESM6_ESM.tiff]

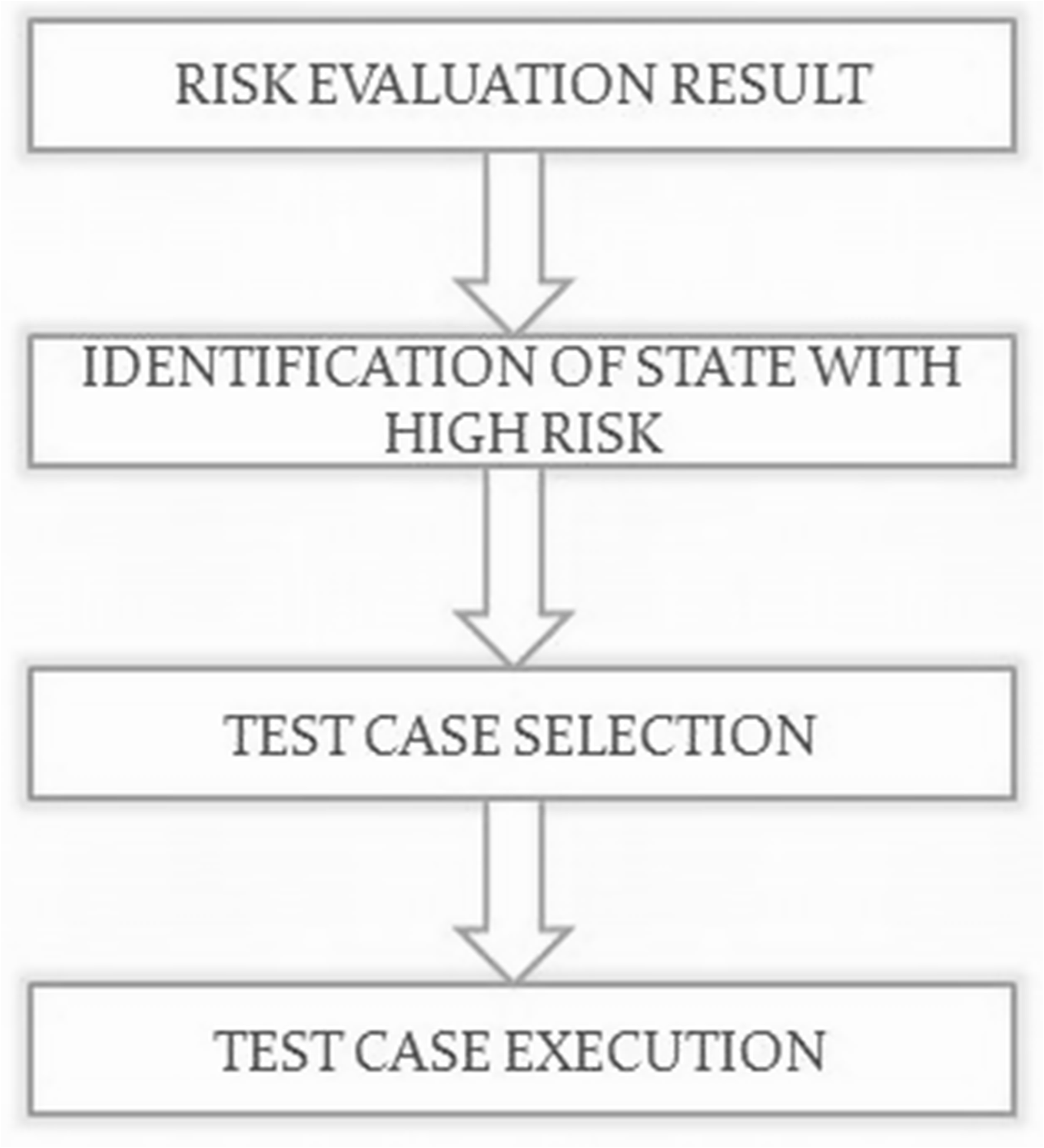

Supplement: Supplementary file 7 — Authors’ original file for figure 7 [file 40064_2014_1515_MOESM7_ESM.tiff]

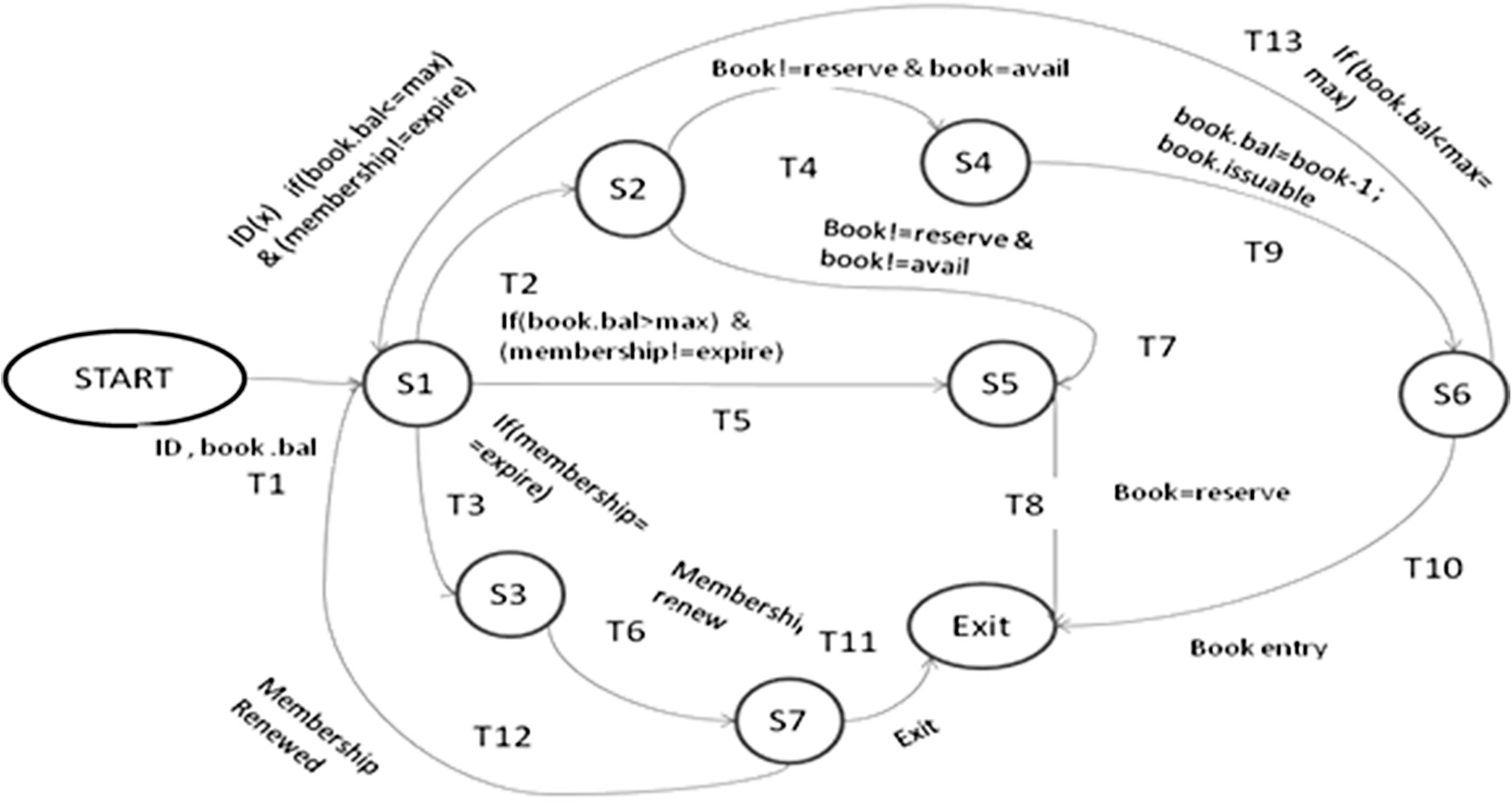

Supplement: Supplementary file 8 — Authors’ original file for figure 8 [file 40064_2014_1515_MOESM8_ESM.tiff]

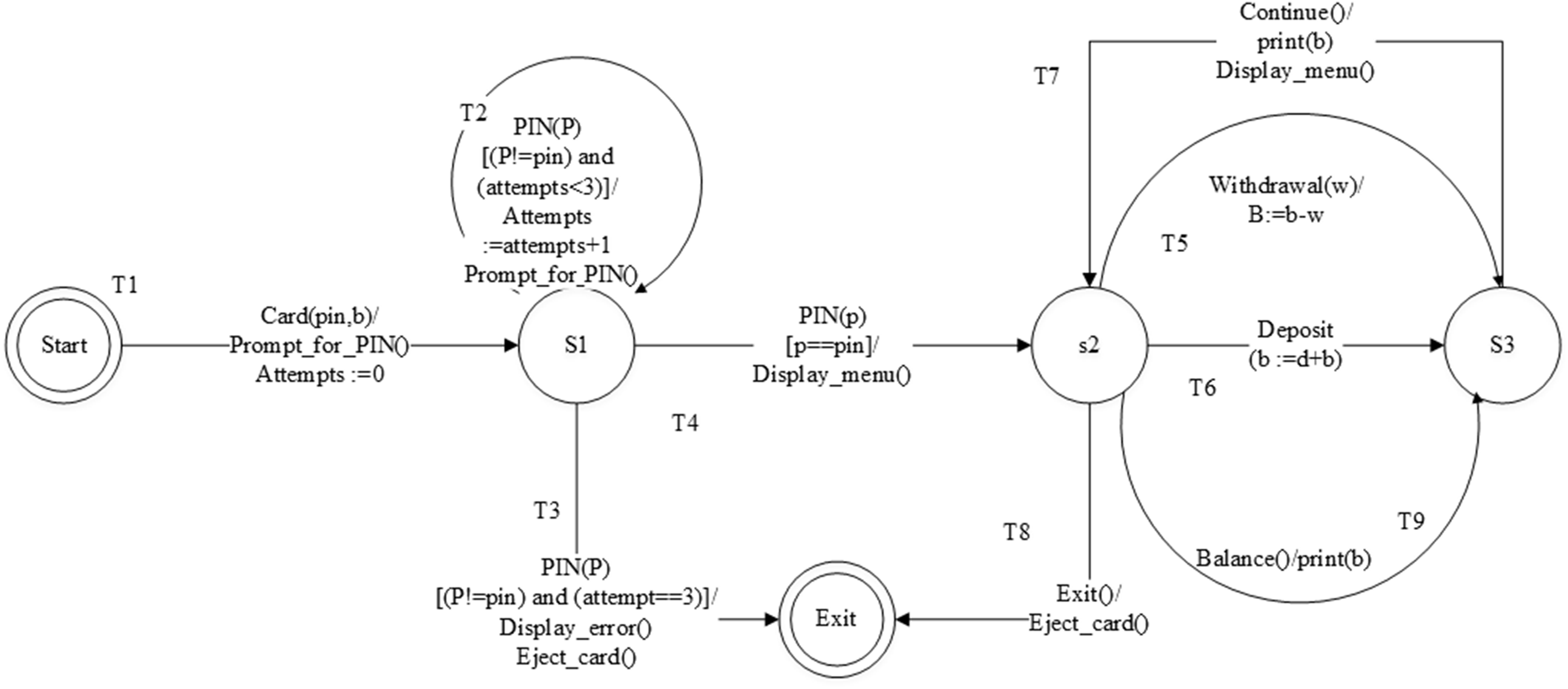

Supplement: Supplementary file 9 — Authors’ original file for figure 9 [file 40064_2014_1515_MOESM9_ESM.tiff]

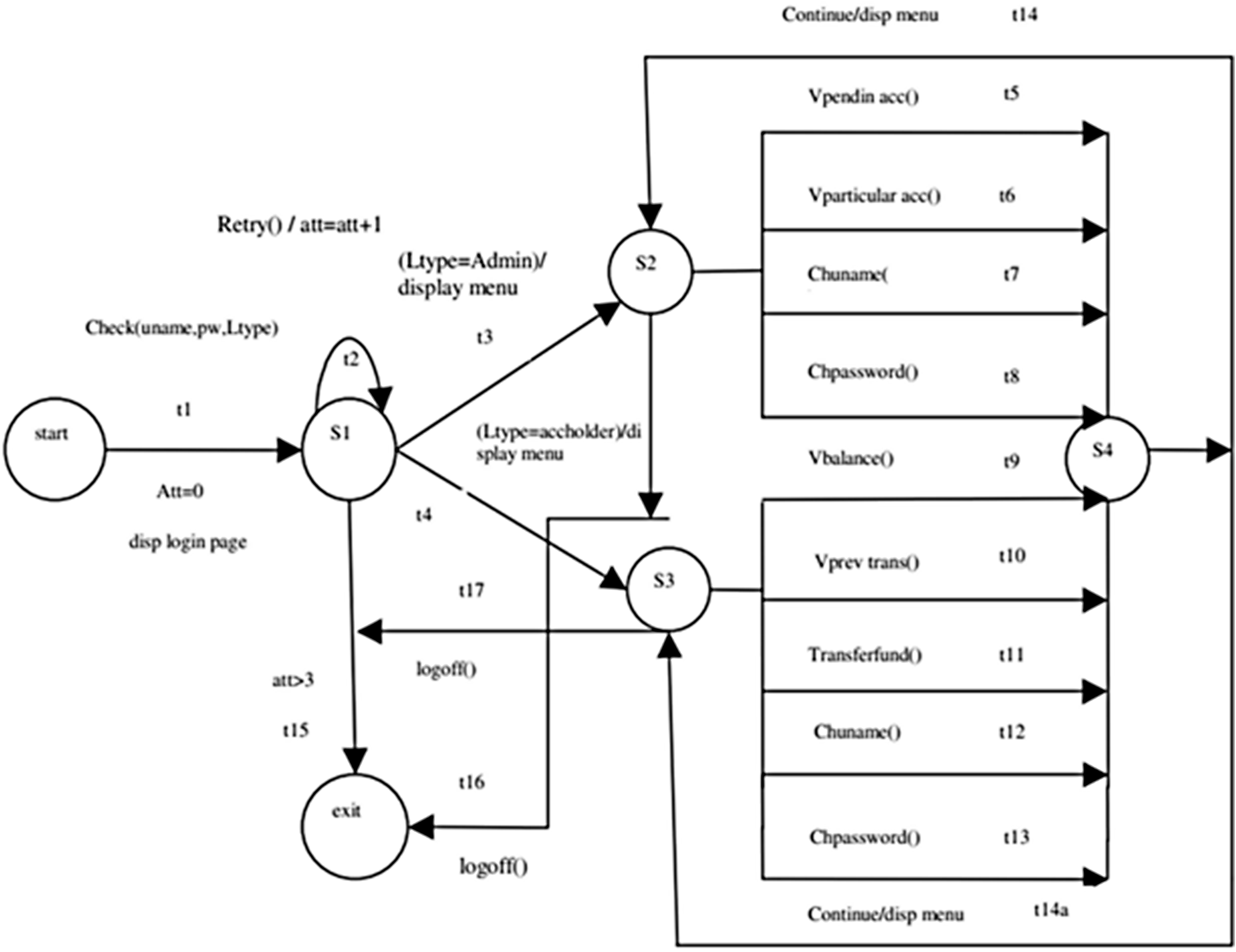

Supplement: Supplementary file 10 — Authors’ original file for figure 10 [file 40064_2014_1515_MOESM10_ESM.tiff]

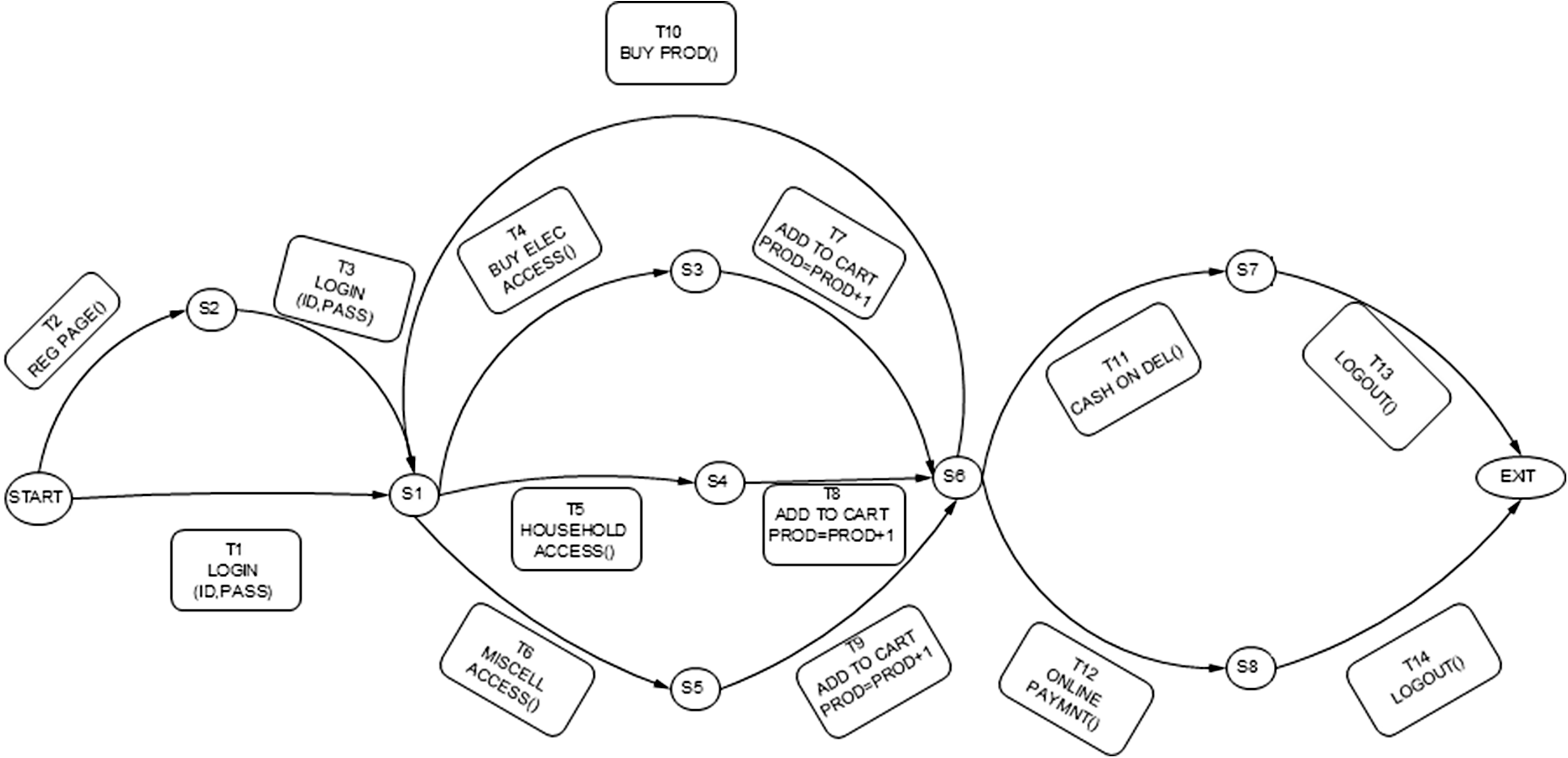

Supplement: Supplementary file 11 — Authors’ original file for figure 11 [file 40064_2014_1515_MOESM11_ESM.tiff]

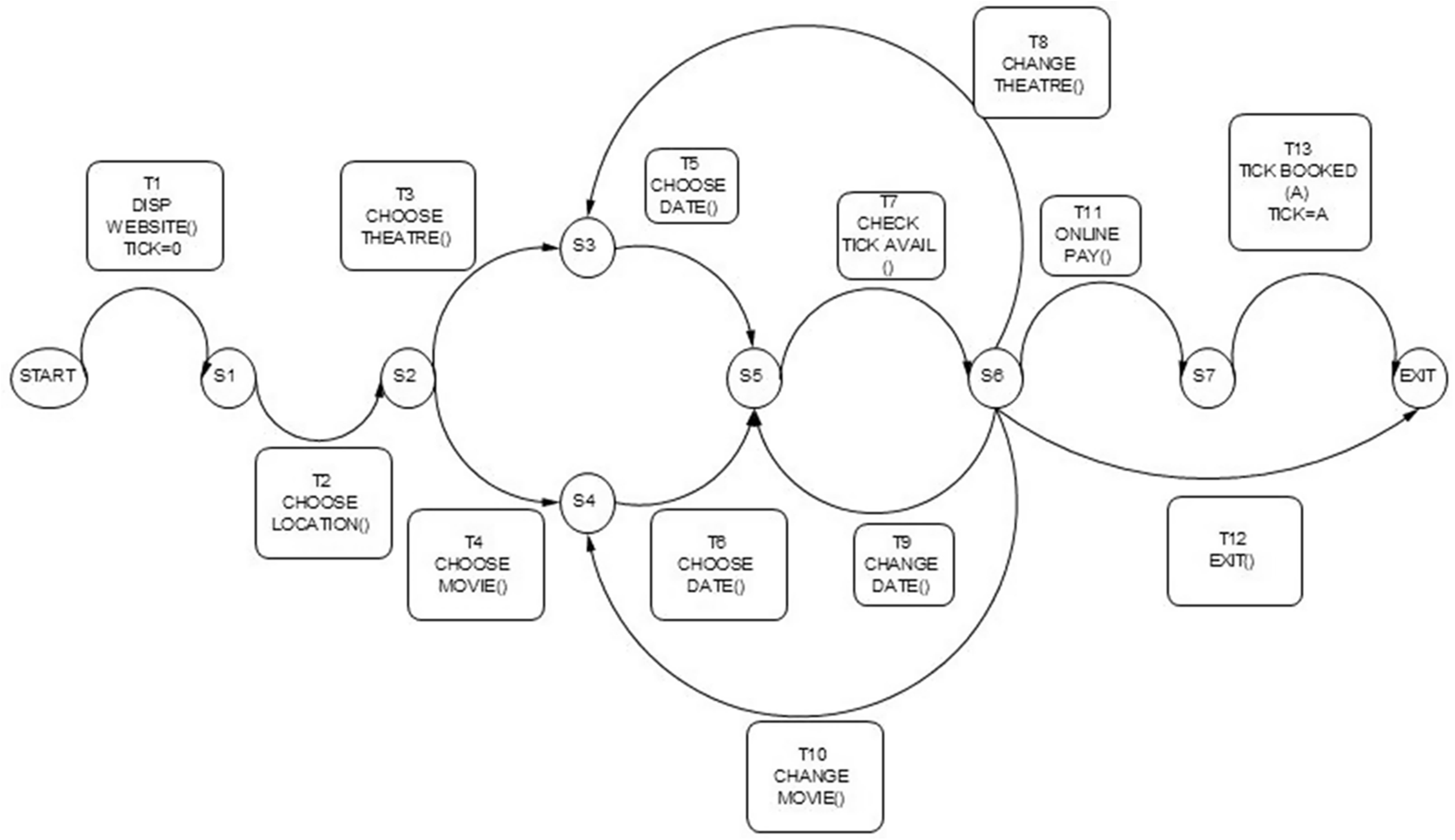

Supplement: Supplementary file 12 — Authors’ original file for figure 12 [file 40064_2014_1515_MOESM12_ESM.tiff]

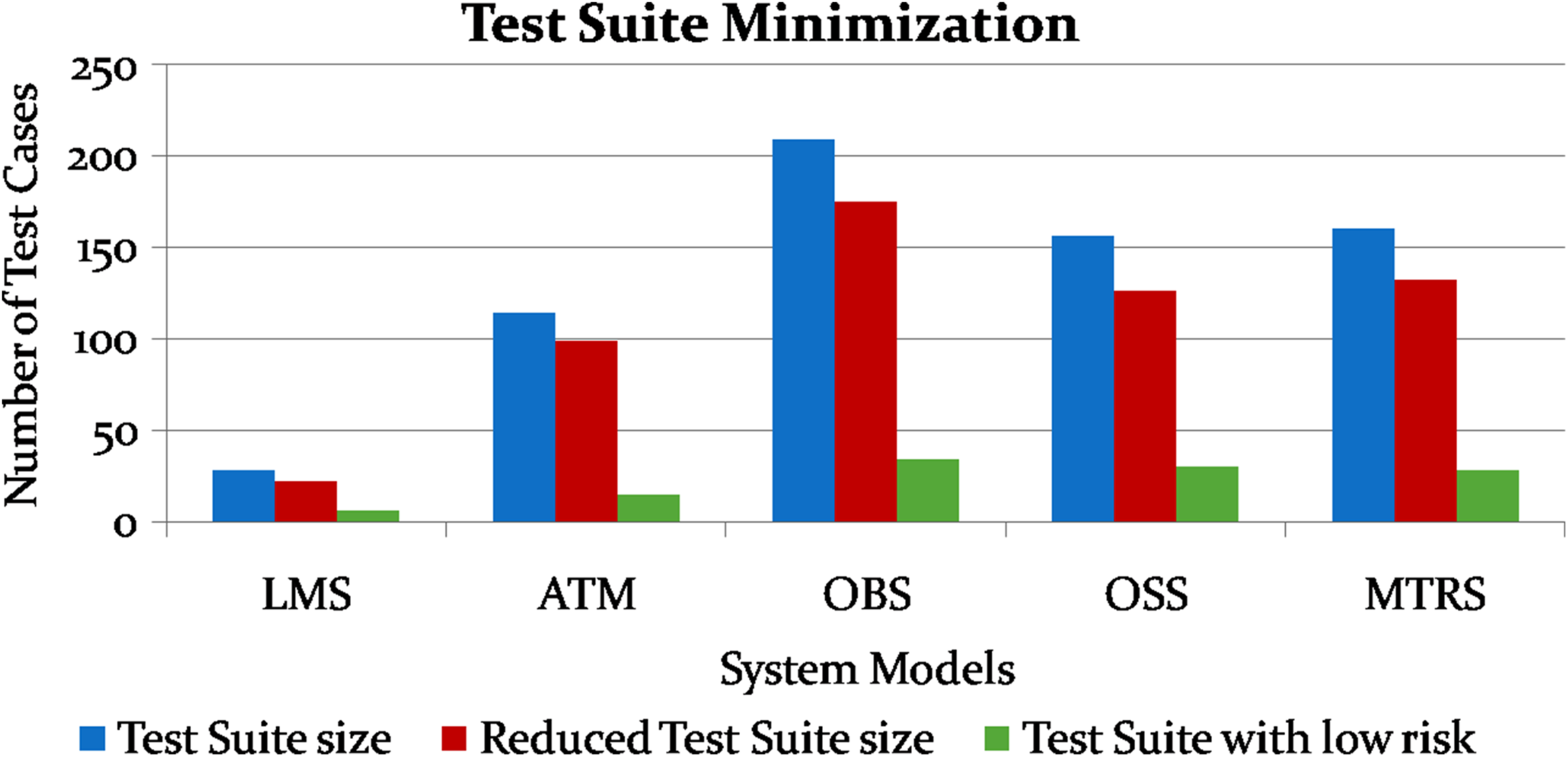

Supplement: Supplementary file 13 — Authors’ original file for figure 13 [file 40064_2014_1515_MOESM13_ESM.tiff]

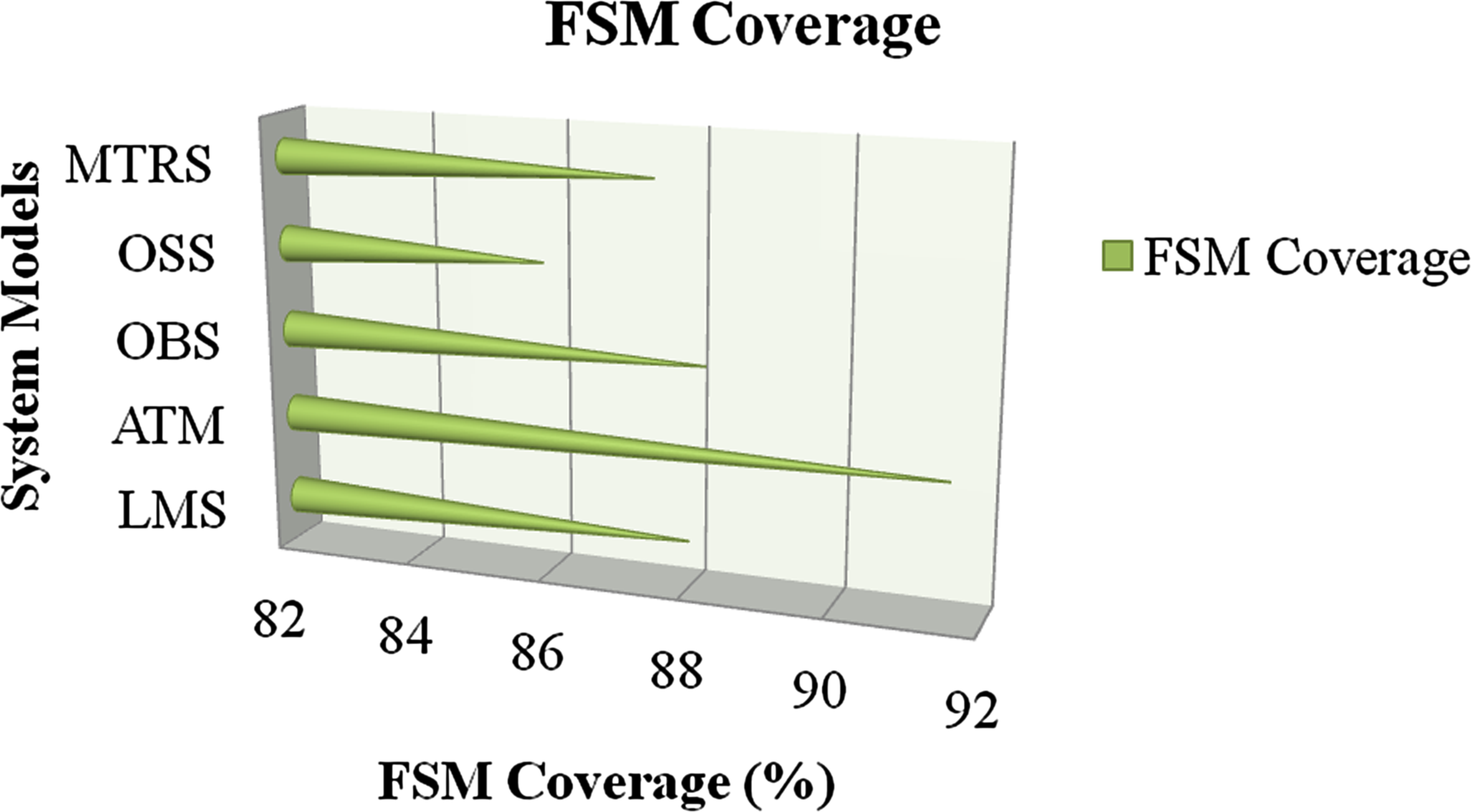

Supplement: Supplementary file 14 — Authors’ original file for figure 14 [file 40064_2014_1515_MOESM14_ESM.tiff]

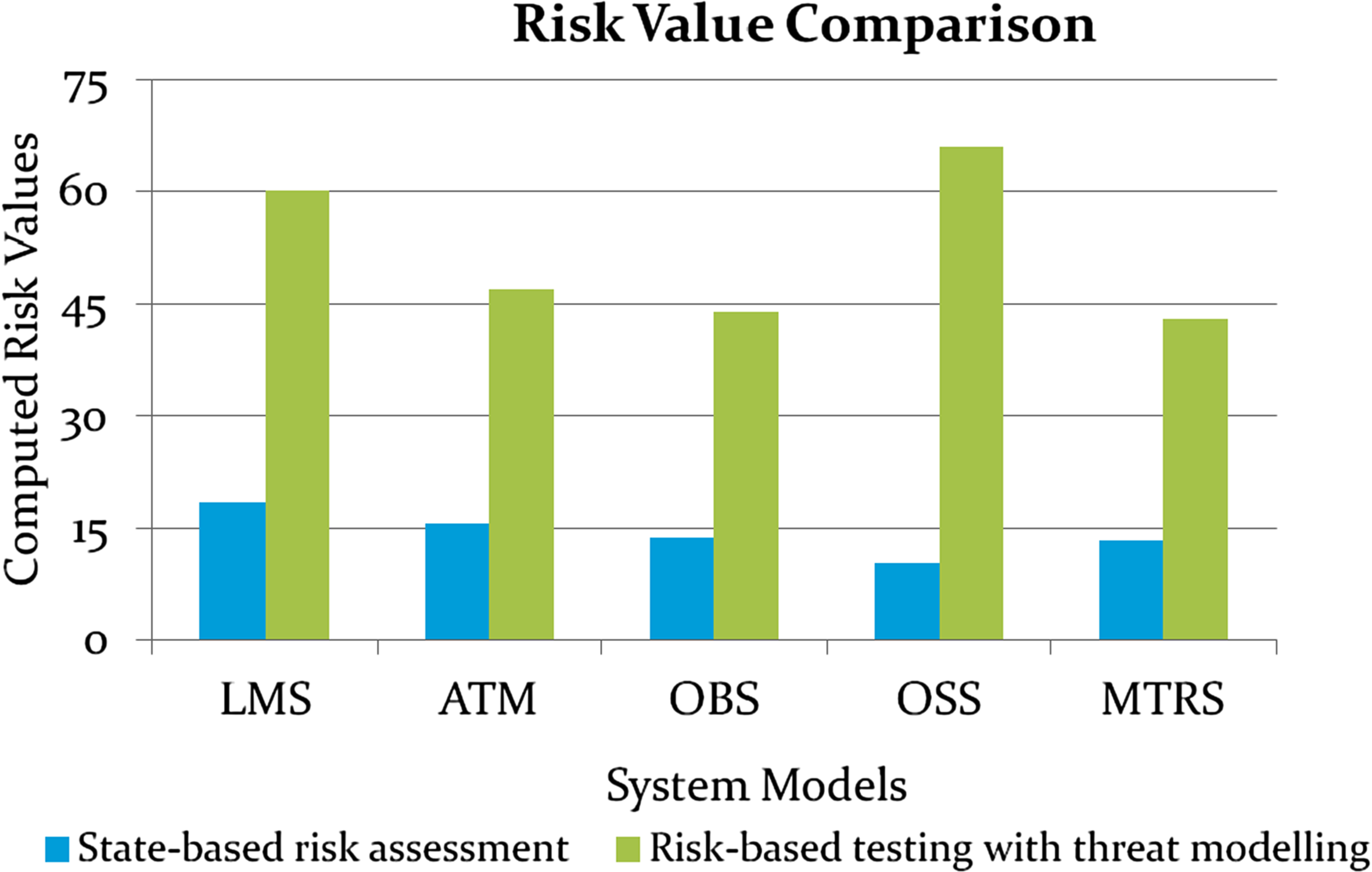

Supplement: Supplementary file 15 — Authors’ original file for figure 15 [file 40064_2014_1515_MOESM15_ESM.tiff]
